# Molecular characterization of novel high-molecular-weight glutenin subunit genes from *Aegilops tauschii*

**DOI:** 10.3389/fpls.2025.1658088

**Published:** 2025-09-19

**Authors:** Huijun Peng, Linlin Lv, Qiuwen Lu, Guojian Zhao, Jiajia Ren, Mian Abdur Rehman Arif, Yarui Su, Ruiru Cheng, Dale Zhang

**Affiliations:** ^1^ State Key Laboratory of Crop Stress Adaptation and Improvement, College of Agriculture, Henan University, Kaifeng, Henan, China; ^2^ School of Life Sciences, Henan University, Kaifeng, China; ^3^ Kaifeng Academy of Agricultural and Forestry Sciences, Kaifeng, China; ^4^ Plant Breeding and Genetics Division, (NIAB), Faisalabad, Pakistan

**Keywords:** *Aegilops tauschii*, high molecular weight glutenin subunit, RNA sequencing, protein secondary structure prediction, SDS-PAGE

## Abstract

The *Glu-D1* locus is known to contribute the most to gluten quality in common wheat. *Aegilops tauschii*, as a donor species of the common wheat D genome, is of great significance for improving wheat flour processing quality by exploring and utilizing its excellent quality-related genes. In this study, high-molecular-weight glutenin subunit (HMW-GS) compositions of 173 *Aegilops tauschii* accessions from the Middle East, Central Asia, and Xinjiang were analyzed using sodium dodecyl sulfate–polyacrylamide gel electrophoresis (SDS-PAGE) technology. A total of 13 subunit types were detected at the G*lu-D1 ^t^* locus, including the unique subunit types absent in common wheat. Unexpectedly, all these *Ae. tauschii* accessions lacked the extra cysteine residue at position 118 in their Glu-1Dx subunits detected by a specific functional marker, *dCAPS5*. To determine whether other sites of Glu-D1^t^ subunits in *Ae. tauschii* contains the extra cysteine residue, the grain RNA of *Ae. tauschii* accessions with rare subunit types at different days post-anthesis (6th, 9th, 12th, 15th, 18th, 21st, and 24th days) were mixed and sequenced using third-generation (PacBio) transcriptome sequencing technology. A total of 12 new HMW-GS genes were obtained, including 6 genes each for *Glu-D1x* and *Glu-D1y*. Sequence alignment analysis revealed the extra cysteine residue was not observed in the protein sequences deduced by 12 new HMW-GS genes. Interestingly, HMW-GS of *Ae. tauschii* accessions AY16 and AY49 had longer repetitive domain insertions, while those of AY16 and AY53 exhibited higher *α*-helix and β-strand contents, which may positively influence dough properties. Additionally, the relative content of HMW-GS in AY11, AY32, and AY49 was significantly higher than that of the *Glu-D1* subunits in wheat variety Xinmai 26 with the strong gluten quality, favoring the formation of larger glutenin polymers. From this point of view, *Ae. tauschii*, as a potentially valuable germplasm resource, can provide rich subunit gene resources for quality breeding in common wheat.

## Introduction

As a donor species of the common wheat D genome, *Aegilops tauschii* (2*n* = 2*x* = 14, DD) contains beneficial genes for disease resistance, insect resistance, and abiotic stress resistance (Sukhwinder et al., 2012; Savary et al., 2019). The Fertile Crescent is considered the origin and genetic diversity center of *Ae. tauschii*, which is widely distributed from west to east along the Caspian Sea coast (Wang et al., 2013). According to morphological characteristics, *Ae. tauschii* is classified as two subspecies, namely, *Ae. tauschii* ssp*. tauschii* and ssp*. strangulata*. Due to that, only a few *Ae. tauschii* genotypes in specific regions (mainly *Ae. tauschii* ssp. *strangulata* subspecies) are involved in the origin of the common wheat D genome, the genetic diversity of *Ae. tauschii* is significantly higher than that of the common wheat D genome (Hao et al., 2020; Gaurav et al., 2022). Therefore, *Ae. tauschii* is considered an important genetic resource for improving common wheat. Some beneficial genes from *Ae. tauschii* have been transferred to common wheat through direct hybridization and indirect methods using tetraploid wheat-*Ae. tauschii* artificial synthetic lines (Zhang et al., 2018, 2017; Zhou et al., 2021; Yang et al., 2022).

The flour processing quality of wheat is significantly correlated with its seed storage proteins (SSPs), which are divided into globulin, albumin, gliadin, and glutenin based on their solubility characteristics (Khalid et al., 2023). Among them, glutenin and gliadin are the main components, accounting for about 90% of the total wheat SSPs. It is generally believed that glutenin, composed of HMW-GS and low-molecular-weight glutenin subunit (LMW-GS), determines the dough elasticity, while gliadin endows the fluidity and extensibility of dough (Guo et al., 2021).

As a structural scaffold for interactions with other glutenin subunits and gliadins, HMW-GS serves as the primary determinant of flour processing quality, critically influencing dough strength and elasticity (Li et al., 2021; Yazar et al., 2017). The HMW-GS of common wheat are composed of a large central repetitive domain flanked by short non-repetitive N- and C-terminal domains, which are named as Ax1 or Ax2^*^ at the *Glu-A1* locus, Bx7 + By8, Bx17 + By18, Bx13 + By16, and Bx14 + By15 at the *Glu-B1* locus, and Dx2 + Dy12 and Dx5 + Dy10 at the *Glu-D1* locus based on their mobility in SDS-PAGE, respectively (Zhang et al., 2016). The *Glu-D1* locus is a key genetic determinant of wheat dough strength and bread-making quality compared with *Glu-A1* and *Glu-B1*. Studies on the contributions of *Glu-1* alleles to flour processing quality have demonstrated that Dx5 and Dy10 subunits were regarded as the most favorable combination (Ma et al., 2005; Blechl et al., 2007; Ren et al., 2022).

Studies have shown that glutenin subunits in *Ae. tauschii* have positive effects on the flour processing quality of wheat (Hsam et al., 2001; Delorean et al., 2021). Moreover, allelic variations of HMW-GS in *Ae. tauschii* exhibit extensive polymorphism, most of which, however, is yet to be discovered in the D genome of common wheat. Therefore, *Ae. tauschii* is regarded as a valuable genetic resource for improving the flour processing quality in wheat (Tahernezhad et al., 2013). In this study, HMW-GS of 173 *Ae. tauschii* accessions from the Middle East, Central Asia, and Xinjiang regions were detected utilizing SDS-PAGE technology. The molecular characterization and relative content of their rare HMW-GS were analyzed, and the results will provide a theoretical basis and genetic resources for wheat quality breeding.

## Materials and methods

### Plant materials

A total of 173 *Ae. tauschii* accessions used in this study were shown in **Supplementary Table S1**. Accessions from the Middle East and Central Asia were provided by the US National Plant Germplasm Center and Institute of Genetics (National Plant Germplasm System, https://www.usda.gov/taxonomy/term/7820), while accessions from Xinjiang were collected and cultivated by the Plant Germplasm Resources and Genetic Engineering Laboratory, Henan University. Common wheat Chinese Spring (null, Bx7 + By8, Dx2 + Dy12) and Xinmai 26 (Ax1, Bx7 + By8, Dx5 + Dy10) were used as standard glutenin subunit controls.

### Glutenin extraction, SDS-PAGE

Glutenin was extracted according to the method described by Zhang et al. (2008a). Half-seeds without embryos were crushed and used for HMW-GS extraction. The flour (20 mg) was first washed with distilled water by stirring for 1h at room temperature to remove the water-soluble proteins. After centrifuging at 5,000*g* for 15 min, the supernatant was discarded and the residue washed with 50% (v/v) propan-1-ol by stirring for 30 min at 60°C. After centrifuging at 5,000*g* for 15 min, the supernatant was discarded. This step was repeated three times to remove gliadins completely. The pellet was extracted with 50% (v/v) propan-1-ol, 0.08 M Tris-HCl buffer (pH 8.0) containing freshly added 1% (w/v) DTT by stirring for 1h at 60 °C. After centrifuging at 12,000*g* for 30 min, the supernatant was precipitated with 1-propanol (final concentration 60% v/v) at 4 °C overnight. The resultant precipitate was collected by centrifuging at 12,000*g* for 15 min and dried by vacuum centrifugation. Samples were separated by SDS-PAGE with a discontinuous system of 4% (w/v) stacking gel and 10% (w/v) separating gel. Eight microliter samples were separately loaded onto lanes of the gel and electrophoresed at approximately 15°C and 12 mA for 18h.

### Genotyping

Genomic DNA was extracted from young leaves according to the approach reported by Su et al. (2020). PCR amplification of the Dx5 subunit in *Ae. tauschii* was performed with marker *dCAPS5* using the method described by Ren et al. (2022) (P1: 5 ‘- GCAGCAAACTCCAACGTA-3’ and P2: 5 ‘- GATAGTATGAAACCTGCTGTCGA-3’). The 25 μl PCR reaction volume contained 50 ng of genomic DNA, 1× Taq DNA polymerase buffer, 0.5 μM of each primer, 200 μM of each dNTP, and 1 U of Taq DNA polymerase (Takara, Japan). The program for PCR amplification was as follows: initial denaturation at 94°C for 3 min, 30 cycles of 94°C for 25 s, 58°C for 25 s, 72°C for 15 s, and a final extension at 72°C for 10 min. The PCR products were digested with restriction enzyme *Sal* I (Takara, Japan). The 10 μl reaction volume consisted of 5 μl of the PCR product, 1 U of the restriction enzyme, and 1× buffer. The reaction was performed at 37°C for 3h, and the digested products were detected with a 3.0% agarose gel. The Dx5 subunit (containing cysteine residues) of the strong-gluten wheat variety Xinmai 26 was used as a control. The amplification products were digested with restriction enzyme *Sal* I, and the digestion products were analyzed by 3% agarose gel electrophoresis.

### PacBio RNA sequencing and data analysis

The timing of anthesis and grain development was inspected and recorded after heading. The grain samples were similarly collected on the 6th, 9th, 12th, 15th, 18th, 21st, and 24th days after flowering (DAF). A total amount of 2 µg RNA was used as input material for the RNA sample preparation. The first-strand cDNA synthesis (reverse transcription and template switching) and PCR amplification were performed utilizing the NEBNext Single Cell/Low Input cDNA Synthesis & Amplification Module (NEB, E6421). HMW-GS genes were obtained through PacBio RNA sequencing and data analysis as described by Ren et al. (2022). Due to the highly complex sequence structure of the HMW-GS genes, a custom pipeline was used to remove redundant sequences to acquire HMW-GS genes in IsoSeq3. Briefly, as queries from the nucleotide sequences of the published glutenin genes in NCBI, the own redundant sequences were first removed utilizing CD-hit software with parameter “-c 0.9.” The obtained nonredundant sequences were merged with those of the annotated glutenin genes in Chinese Spring (RefSeq v1.0) to form query sequences. Then, Blat software (Kent, 2002) was used to search for the FLNC sequence generated by IsoSeq3 based on the query sequences, in which those with coverage and identity greater than 70% were retained, followed by error correction and removal of redundant reads using IsoCon software (Sahlin et al., 2018). Thus, obtained sequences were aligned by MAFFT software (Katoh and Standley, 2013) and manually removed again in redundant ones according to the support score acquired by IsoCon.

### Sequence alignment and secondary structure prediction

The open reading frame (ORF) of the target gene was translated into an amino acid sequence using the NCBI ORF Finder program (http://www.ncbi.nlm.nih.gov). The alignment of sequences was carried out using the Clustal X 2.0 software (Larkin et al., 2007). Secondary structure prediction of deduced amino acid sequences was performed by PSIPRED server 4.0 (http://bioinf.cs.ucl.ac.uk/psipred/). Default parameters were used in these analyses.

### Detection of glutenin subunit relative quantities

Equal weights (100 mg each) of sieved flour from *Ae. tauschii* accessions, Xinmai 26, and Chinese Spring were used for glutenin extraction following the method described by Zhang et al. (2016). The extracted glutenin was separated by SDS-PAGE with 20 μl per sample and visualized by Coomassie Blue staining. Bovine serum albumin (BSA) at 0.4 μg/μl was used as a reference control. Densitometric analyses of glutenin subunits were performed using Image J software (Bio-Rad, http://www.bio-rad.com). The value of the optical density multiplication area was used to quantify HMW-GS abundance. Student’s t-test was used to determine the significance of differences (*P* < 0.05) between *Ae. tauschii* and Xinmai 26.

## Results and discussion

### Composition and allelic variation of HMW-GS in 173 *Ae. tauschii* accessions

**Figure 1:**
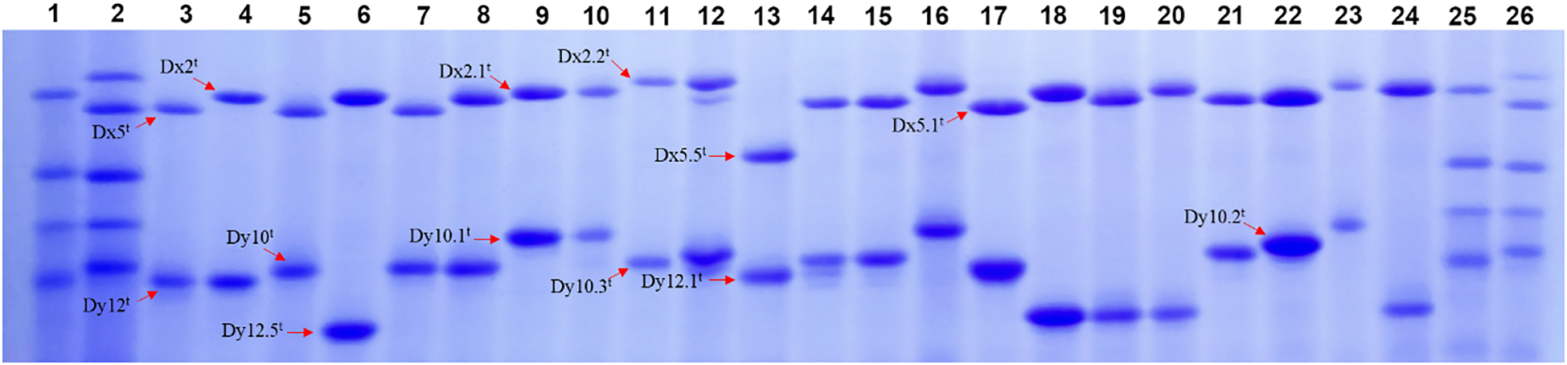
SDS-PAGE patterns of HMW-GS in partial *Ae. tauschii* accessions. (1, Chinese spring; 2, Xinmai 26; 3, AY1; 4, AY34; 5, AY27; 6, AY6; 7, AY33; 8, AY23; 9, AY79; 10, AY37; 11, AY73; 12, AY9; 13, AY32; 14, AY38; 15, AY15; 16, AY11; 17, AY16; 18, AY60; 19, AY55; 20, AY61; 21, AY8; 22, AY29; 23, AY24; 24, AY53;25, Chinese spring; 26, Xinmai 26).

HMW-GS compositions of 173 *Ae. tauschii* accessions were analyzed based on their mobility in SDS-PAGE, with Chinese Spring (null, Bx7 + By8, Dx2 + Dy12) and Xinmai 26 (Ax1, Bx7 + By8, Dx5 + Dy10) as standards (**Figure 1**; **Supplementary Table S1**; **Supplementary Figure S1**). The results showed that HMW-GS combinations of *Ae. tauschii* exhibited high diversity, and a total of 16 distinct types were identified. The most frequent subunit combination was Dx2^t^ + Dy10^t^, accounting for 68.2% of accessions, followed by Dx5^t^ + Dy10^t^ (13.3%). The other subunit combinations included Dx5^t^ + Dy12^t^, Dx2.1^t^ + Dy10.1^t^, Dx5^t^ + Dy12.5^t^, Dx5.5^t^ + Dy12.1^t^, Dx2.2^t^ + Dy10^t^, Dx2^t^ + Dy10.2^t^, Dx2^t^ + Dy10.1^t^, and Dx2^t^ + Dy12.5^t^, and so forth. Allelic variation and subunit frequency analysis at the *Glu-Dx1* locus revealed that the Dx2^t^ subunit has the highest frequency (76.3%), followed by the Dx5^t^ subunit (16.7%) (**Table 1**). In addition, several rare subunits in common wheat were detected, including Dx2.1^t^, Dx2.2^t^, Dx5.1^t^, and Dx5.5^t^, with frequencies of 2.3%, 2.3%, 1.2%, and 1.2%, respectively. At the *Glu-Dy1* locus, Dy10^t^ was the most prevalent subunit (83.2%), while Dy12^t^ occurred at 5.8%. Five rare subunits in common wheat were also identified, including Dy10.1^t^, Dy10.2^t^, Dy10.3^t^, Dy12.1^t^, and Dy12.5^t^, with frequencies of 2.9%, 2.3%, 1.2%, 1.2%, and 3.4%, respectively.

**Table 1:** HMW-GS allelic variations and frequencies in 173 *Ae. tauschii* accessions.

Locus	Subunit	Accessions	Frequency (%)
Glu-D1	Dx2^t^	132	76.3
	Dx2.1^t^	4	2.3
	Dx2.2^t^	4	2.3
	Dx5^t^ Dx5.1^t^	29 2	16.7 1.2
	Dx5.5^t^	2	1.2
	Dy10^t^ Dy10.1^t^	144 5	83.2 2.9
	Dy10.2^t^	4	2.3
	Dy10.3^t^	2	1.2
	Dy12^t^ Dy12.1^t^	10 2	5.8 1.2
	Dy12.5^t^	6	3.4

The Dx5 + Dy10 subunit combination is considered a superior type for bread-making quality in wheat. As the donor species of the common wheat D genome, *Ae. tauschii* possesses more diverse HMW-GS combinations and allelic variation at the *Glu-D1* locus. Tahernezhad et al.^19^ identified eight novel subunit combinations in 28 *Ae. tauschii* accessions from Iran, including Dx1.5^t^ + Dy10^t^, Dx3^t^ + Dy11^t^, Dx2^t^ + Dy10.1^t^, Dx3^t^ + Dy12^t^, Dx3^t^ + Dy12.3^t^, Dx1.5^t^ + Dy12^t^, Dx3^t^ + Dy12.2^t^, and Dx1.5^t^ + Dy12.1^*t^. These results demonstrate that HMW-GS compositions of *Ae. tauschii* has abundant genetic diversity, which could provide valuable subunit gene resources for common wheat quality breeding.

It is widely accepted that the Dx5 subunit, as a superior high-quality strong glutenin subunit, has been extensively utilized in wheat quality breeding since it contains an extra cysteine residue (codon: TGT) at the 118th position of the amino acid sequence. This residue can form additional disulfide bonds with other HMW-GS or LMW-GS, generating larger glutenin polymers that positively influence dough viscoelasticity (Sun et al., 2024). It is natural to know whether the extra cysteine residue in wheat Dx5 subunit directly originates from *Ae. tauschii*. To address this issue, 173 *Ae. tauschii* accessions were analyzed utilizing the specific functional marker *dCAPS5*, which was developed to target the extra cysteine residue in the wheat Dx5 subunit (Ren et al., 2022). Among them, accessions AY01, AY06, AY08, AY09, AY16, AY40, AY49, AY55, AY60, AY61, and AY80 belong to the L2E family (Li et al., 2022), which has been identified as the direct donor of the wheat D genome (Wang et al., 2013, 2024; Cavalet-Giorsa et al., 2024). Unexpectedly, none of the *Ae. tauschii* accessions carried the extra cysteine residue at position 118 in their Dx5^t^ subunits (**Supplementary Table S1**). Similar results were reported by Dong et al. (2013), in which none of the Glu-Dx5^t^ subunits with the extra cysteine residue at position 118 were found in 355 *Ae. tauschii* accessions from over 20 countries using seven molecular markers linked to the wheat Dx5 subunit. Additionally, alignment analysis of the amino acid sequence deduced by the cloned Dx5^t^ subunit gene showed that none of the extra cysteine residue was observed except for four conserved cysteine residues (Yan et al., 2008; Zhang et al., 2008b). Furthermore, Pflüger et al. (2001) found that the two subunits exhibited significant differences in surface hydrophobicity compared with *Ae. tauschii* Dx5^t^ subunit and wheat Dx5 subunit using reverse-phase high-performance liquid chromatography (RP-HPLC), attributing this difference primarily to the extra cysteine residue in the wheat Dx5 subunit. All these findings demonstrated that the wheat Dx5 subunit might not originate directly from theDx5^t^ subunit in *Ae. tauschii*. Wang et al. (2024) proposed that the wheat D genome predominantly accumulated novel mutations derived from *Ae. tauschii* rather than direct introgression through analyzing genomic structural variations of 292 wheat and 400 *Ae. tauschii* accessions, where the relative importance of novel mutation accumulation was much higher for the genetic diversity of the D genome. This contrast can be partially attributed to frequent interploidy introgression from tetraploid wheat into hexaploid wheat, while rare introgressions from *Ae. tauschii* to hexaploid wheat. The multi-layered genetic diversity of the D genome was caused by the accumulation of variations, and key genetic loci such as *TaBTR-D1*, *TaPPD-D1*, and *TaGlu-D1* acquired genetic diversity through this featured structure, thereby facilitating the adaptation and agronomic utilization of hexaploid wheat (Wang et al., 2024). Thus, current evidence suggests the extra cysteine residue in wheat 1Dx5 might arise from mutational events during the evolutionary process from *Ae. tauschii* to common wheat.

### Molecular characterization of HMW-GS genes from *Ae. tauschii*

To determine whether other sites of Glu-D1^t^ subunits in *Ae. tauschii* contain the extra cysteine residue except for the Dx5^t^ subunit; the accessions AY11, AY16, AY29, AY32, AY49, AY53, and AY73, containing all subunits (Dx2^t^, Dx2.1^t^, Dx2.2^t^, Dx5^t^, Dx5.1^t^, Dx5.5^t^, Dy10^t^, Dy10.1^t^, Dy10.2^t^, Dy10.3^t^, Dy12^t^, Dy12.1^t^, and Dy12.5^t^) in this study, were selected for the third-generation transcriptome sequencing through the PacBio Sequel II platform. A total of 164,696,323 subreads with a mean length of 1,818 bp were generated, from which the 3,358,335 reads of a circular consensus sequence (CCS) were extracted. The 3,162,107 full-length nonchimeric sequences (FLNCs) were acquired after refining by the IsoSeq3 pipeline. Due to the highly complex sequence structure of HMW-GS, the redundant sequences were further removed utilizing a custom pipeline in FLNCs. Thus, the obtained sequences were further aligned, and manually removed again in redundant ones. Finally, fourteen transcripts of HMW-GS genes were identified based on the alignment with the published glutenin genes from *Triticeae* (**Table 2**; **Supplementary Table S2**).

**Table 2:** Fourteen transcripts of HMW-GS identified in *Ae. tauschii* accessions.

Accessions	Subunits	Transcript ID	Nucleotide (bp)	Signal peptide	N-terminal domain	Repetitive domain	C-terminal domain
AY49	Dx5^t^	*transcript66*	2568	21	89(3)	702	42(1)
AY53	Dx2^t^	*transcript370*	2541	21	89(3)	693	42(1)
AY29	Dx2^t^	*transcript506*	2541	21	89(3)	693	42(1)
AY32	Dx5.5^t^	*transcript533*	2115	21	89(3)	551	42(1)
AY73	Dx2.2^t^	*transcript720*	2487	21	89(3)	675	42(1)
AY16	Dx5.1^t^	*transcript780*	2568	21	89(3)	702	42(1)
AY11	Dx2.1^t^	*transcript928*	2514	21	89(3)	684	42(1)
AY49	Dy12^t^	*transcript51*	1962	21	104(5)	485(1)	42(1)
AY11	Dy10.1^t^	*transcript67*	1995	21	104(5)	496(1)	42(1)
AY53	Dy12.5^t^	*transcript361*	1878	21	104(5)	457(1)	42(1)
AY29	Dy10.2^t^	*transcript447*	1986	21	104(5)	493(1)	42(1)
AY16	Dy10^t^	*transcript637*	1980	21	104(5)	491(1)	42(1)
AY32	Dy12.1^t^	*transcript690*	1950	21	104(5)	481(1)	42(1)
AY73	Dy10.3^t^	*transcript754*	1968	21	104(5)	487(1)	42(1)

The values in parentheses indicate the number of amino acids.

Sequence alignment with published HMW-GS genes in GenBank revealed that *transcript928* and *transcript67* showed complete identity to the previously reported Dx subunit gene (Accession: AF480486) and Dy subunit gene (Accession: FJ008134), respectively. Consequently, six novel Dx subunit genes (*transcript720, transcript66, transcript533, transcript370, transcript506*, and *transcript780*) and six novel Dy subunit genes (*transcript637, transcript51, transcript361, transcript447, transcript754*, and *transcript690*) were identified. All these *Ae. tauschii* HMW-GS gene sequences initiated with the start codon ATG and terminated with the double stop codon TGATAG.

**Figure 2:**
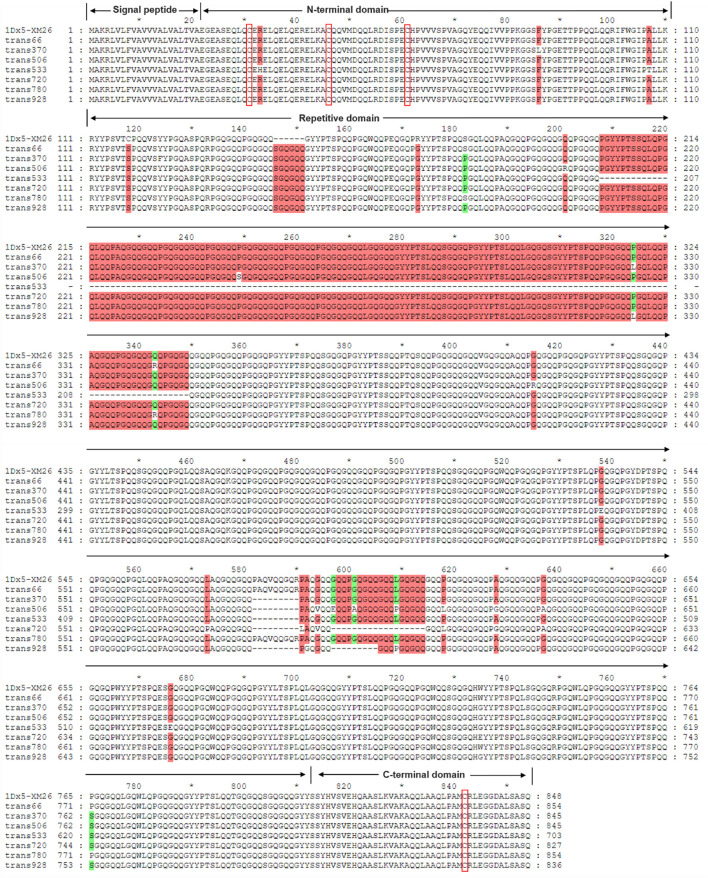
Sequence alignment of 7 identified Dx subunits in *Ae. tauschii* and the Dx5 subunit in Xinmai 26. (The red boxes indicate the conserved cysteine residues).

Sequences of the seven identified Dx subunit genes ranged from 2115 to 2568 bp in length, encoding 703–854 amino acids (**Table 2**; **Supplementary Table S2**). Notably, the 1Dx5.5^t^ subunit exhibited a 426-bp deletion in the central repetitive domain, resulting in the shortest variant of only 703 amino acids. Sequence alignment analysis with the reference Dx5 subunit in wheat variety Xinmai 26 with the strong gluten demonstrated that all seven Dx subunits in *Ae. tauschii* only contained four conserved cysteine residues located at positions 10, 25, and 40 in the N-terminal domain and position 30 in the C-terminal domain (**Figure 2**). The central repetitive domains (containing 551 to 702 amino acids) emerged as the primary source of molecular diversity among these subunits. Particularly, the Dx5.1^t^ subunit of AY16 and the Dx5^t^ subunit of AY49 had a six-amino-acid insertion (SGQGQQ) at positions 146–151 within the central repetitive region. Compared with other subunits, the two subunits displayed higher sequence similarity with the high-quality subunit Dx5, implying their potential values for wheat quality improvement.

The full lengths of seven Dy subunit genes ranged from 1878 to 1995 bp, encoding 624 to 663 amino acids (**Table 2**). The positions of the seven cysteine residues contained in the Dy subunit sequence are very conserved, with five in the N-terminal domain (at positions 10, 22, 44, 45, and 55), one in the central repetitive domain (at position 418), and one in the C-terminal domain (at position 32) (**Supplementary Figure S2**). Compared with the Dy10 subunit in wheat variety Xinmai 26, the 1Dy10^t^ subunit in AY16 has two identical six-peptide insertions (QIGQGQ) at positions 202–207 and 229–234 within its central repetitive region (**Supplementary Figure S2**). The longer repetitive region may have positive effects on dough properties and consequently possess high potential for wheat quality improvement (Wang et al., 2012).

The secondary structure of HMW-GS, primarily composed of α-helices and β-strands in wheat gluten, forms the structural foundation for its complex spatial conformation. According to Masci et al. (1998), helix–helix interactions were critical for intramolecular disulfide bond formation. The increased α-helix content is likely to play a significant role in enhancing dough quality, and the β-strands are believed to be positively correlated with dough elasticity and resistance to distortion capability (Jiang et al., 2008). The secondary structures of all subunits in this study were predicted and compared with previously reported Glu-D1 subunits of wheat Xinmai 26 with strong gluten (**Table 3**). As a result, the α-helices of HMW-GS in *Ae. tauschii* and Xinmai 26 were predominantly localized in both N-terminal and C-terminal domains, while β-strands were exclusively confined to the N-terminal domains. The Dx2^t^ subunit of AY53 exhibited significantly higher α-helix and β-strand content than the Dx5 subunit of Xinmai 26, containing 65 and 11 amino acid residues, respectively. The Dy subunits of *Ae. tauschii* generally have fewer β-strands, with the Dy subunits of AY11, AY29, AY53, and AY73 even lacking β-strands. In comparison, the Dy10^t^ subunit of AY16 shows significantly higher α-helix and β-strand content than those of Xinmai 26, containing 70 and 2 amino acid residues, respectively.

**Table 3:** Secondary structure prediction of HMW-GS in *Ae. tauschii*.

							Dispersal in every region		
Accessions	Subunits	Sequence ID	Structure motifs	Amino acid number	Ratio(%)	Total	N-terminal domain	Repetitive domain	C-terminal domain
AY11	Dx2.1^t^	*transcript928*	α-helix	60	7.36	4	2	–	2
			β-strand	13	1.60	5	5	–	–
AY16	Dx5.1^t^	*transcript780*	α-helix	60	7.36	4	2	–	2
			β-strand	11	1.32	3	3	–	–
AY29	Dx2^t^	*transcript370*	α-helix	60	7.28	4	2	–	2
			β-strand	13	1.58	2	2	–	–
AY32	Dx5.5^t^	*transcript533*	α-helix	60	8.80	4	2	–	2
			β-strand	2	0.29	1	1	–	–
AY49	Dx5^t^	*transcript66*	α-helix	59	7.07	4	2	–	2
			β-strand	11	1.32	4	4	–	–
AY53	Dx2^t^	*transcript506*	α-helix	65	7.69	4	3	–	1
			β-strand	11	1.33	4	4	–	–
AY73	Dx2.2^t^	*transcript720*	α-helix	59	7.32	4	2	–	2
			β-strand	10	1.24	4	4	–	–
Xinmai 26	Glu-Dx5	*Dx5*	α-helix	60	7.26	4	2	–	2
			β-strand	12	1.45	3	3	–	–
AY11	Dy10.1^t^	*transcript67*	α-helix	68	10.59	6	3	–	3
AY16	Dy10^t^	*transcript637*	α-helix	70	11.09	6	3	–	3
			β-strand	2	0.32	1	1	–	–
AY29	Dy10.2^t^	*transcript447*	α-helix	67	10.49	6	3	–	3
AY32	Dy12.1^t^	*transcript690*	α-helix	61	9.73	6	3	–	3
			β-strand	2	0.32	1	1	–	–
AY49	Dy12^t^	*transcript51*	α-helix	66	10.46	6	3	–	3
			β-strand	3	0.48	1	1	–	–
AY53	Dy12.5^t^	*transcript361*	α-helix	72	11.94	6	3	–	3
AY73	Dy10.3^t^	*transcript754*	α-helix	66	10.43	6	3	–	3
Xinmai 26	Glu-Dy10	*Dy10*	α-helix	66	10.53	6	3	–	3
			β-strand	4	0.64	1	1	–	–

The results of HMW-GS sequence composition and secondary structure content revealed that the Dx5.1^t^ + Dy10^t^ subunit combination of accession AY16 possesses the longer repetitive domain and higher α-helix and β-strand content compared to the other *Ae. tauschii* accessions, which makes it potentially a valuable germplasm resource for improving wheat quality.

### Relative content of HMW-GS in *Ae. tauschii*

**Figure 3:**
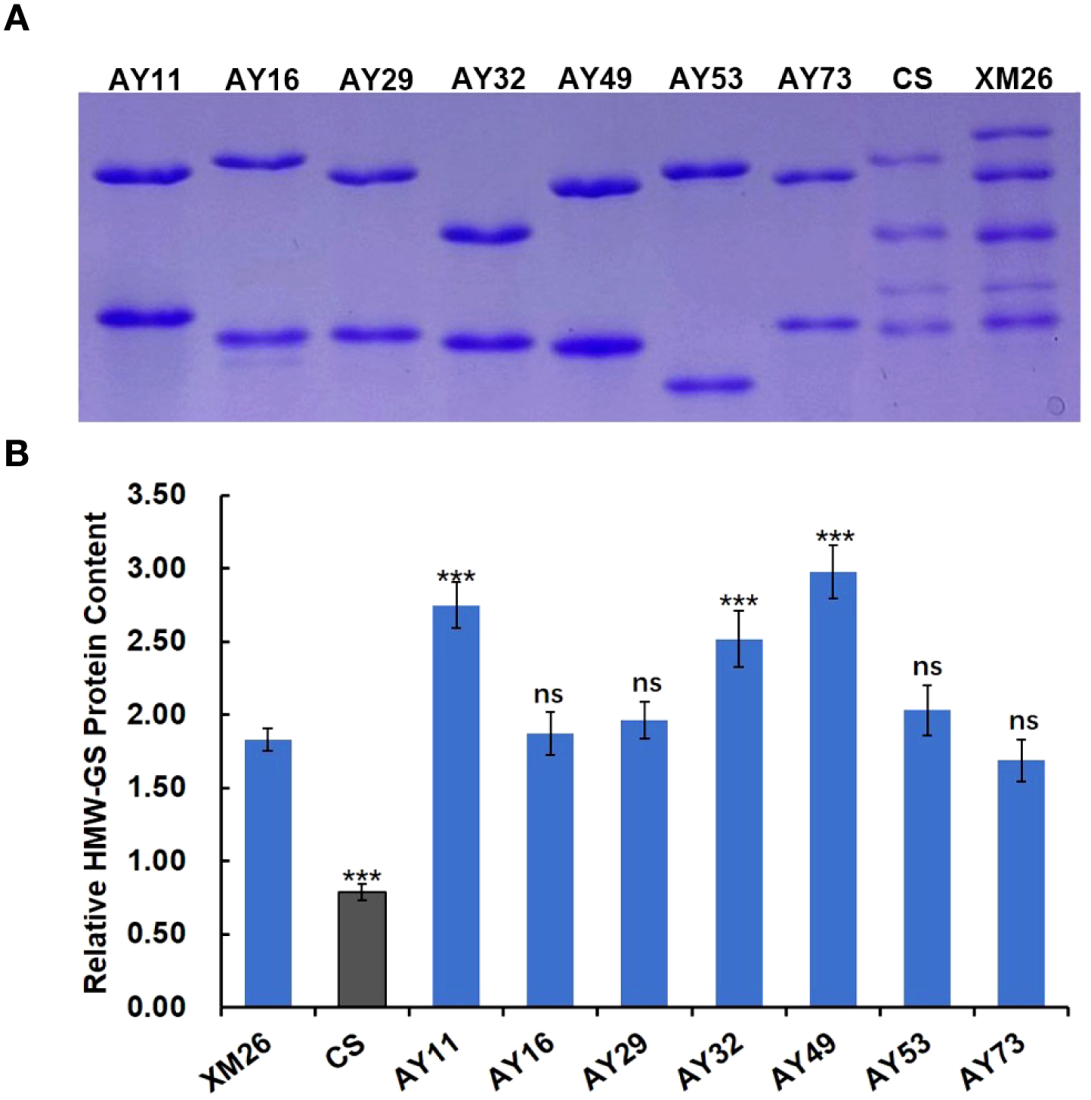
HMW-GS content in AY11, AY16, AY29, AY32, AY49, AY53, AY73, Xinmai 26 and Chinese Spring. (**A**, electrophoresis patterns of HMW-GS in the same amount of flour. **B**, the relative content of HMW-GS. XM26, Xinmai 26; CS, Chinese Spring; ns, no significance. ***indicate significantly different from Xinmai 26 at *P* = 0.001).

The viscoelastic properties of dough are influenced not only by glutenin subunit composition but also by glutenin content in the grain. Higher glutenin content can promote the formation of larger glutenin macropolymers, which in turn stabilizes the structural integrity and enhances the dough elasticity (Gao et al., 2021; Yang et al., 2023). The HMW-GS of 7 *Ae. tauschii* accessions (AY11, AY16, AY29, AY32, AY49, AY53, and AY73) were separated by SDS-PAGE, with Xinmai 26 and Chinese Spring as controls at equivalent amounts. As shown in **Figure 3A**, the band of the most *Ae. tauschii* accessions displayed significantly larger volume and more intense staining than Xinmai 26. This reveals that the content of HMW-GS in these *Ae. tauschii* accessions are higher than the Glu-D1 subunit in common wheat.

The relative content of HMW-GS in *Ae. tauschii* and wheat was quantified based on optical density using Image J software, with BSA as the reference protein (**Figure 3b**). The results showed that relative HMW-GS content in all *Ae. tauschii* accessions exhibited higher than that in Chinese Spring. Notably, the relative content of HMW-GS in AY11, AY32, and AY49 was significantly higher than that of the Glu-D1 subunits in the wheat variety Xinmai 26 with strong gluten, which indicated that these three accessions could be valuable germplasm resources for wheat quality improvement.

In conclusion, HMW-GS compositions of *Ae. tauschii* exhibited remarkable genetic diversity. However, none of the Glu-D1^t^ subunits in *Ae. tauschii* contained the extra cysteine residue at position 118, suggesting that the extra cysteine of the Dx5 subunit in wheat might have arisen from mutational events during the evolution of hexaploid wheat from its progenitors. Additionally, the longer repetitive domains and higher α-helix and β-strand contents in HMW-GS of *Ae. tauschii* may have positive effects on dough properties. Furthermore, the higher HMW-GS content in some *Ae. tauschii* accessions, such as AY11, AY32, and AY49, may facilitate the formation of larger glutenin macropolymers. Therefore, *Ae. tauschii* could be used as a valuable germplasm resource for enriching the genetic basis of wheat quality breeding.

## Data Availability

All raw data of the PacBio RNA sequencing in this study were deposited in the National Center for Biotechnology Information (NCBI) under BioProject number PRJNA1307214.
