# Supplementary material for: Molecular characterization of novel high-molecular-weight glutenin subunit genes from Aegilops tauschii
Source: Front Plant Sci. 2025 Sep 19;16:1658088. doi: 10.3389/fpls.2025.1658088 (PMC12491290; doi:10.3389/fpls.2025.1658088)
Supplement: Supplementary file 1 [file DataSheet1.docx]

**Supplementary Material**

**Molecular characterization of novel high molecular weight glutenin subunit genes from *Aegilops tauschii***

Huijun Peng^1,2+^, Linlin Lv^1,2+^, Qiuwen Lu^2^, Guojian Zhao^3^, Jiajia Ren^3^, Mian Abdur Rehman Arif^4^, Yarui Su^1^, Ruiru Cheng^1^*, Dale Zhang^1^*

Table S1 Information of 173 *Ae. tauschii* accessions.

| Number | Accession | Origin | Subunit combination | *dCAPS5* ^a^  (76/95bp) |
| --- | --- | --- | --- | --- |
| 1 | XJ1 | Huocheng, Xinjiang | 2^t^ +10^t^ | - |
| 2 | XJ2 | Huocheng, Xinjiang | 2^t^+10^t^ | - |
| 3 | XJ3 | Huocheng, Xinjiang | 2^t^+10^t^ | - |
| 4 | XJ4 | Huocheng, Xinjiang | 2^t^+10^t^ | - |
| 5 | XJ5 | Huocheng, Xinjiang | 2^t^+10^t^ | - |
| 6 | XJ6 | Huocheng, Xinjiang | 2^t^+10^t^ | - |
| 7 | XJ7 | Huocheng, Xinjiang | 2^t^+10^t^ | - |
| 8 | XJ12 | Huocheng, Xinjiang | 5^t^+10^t^ | - |
| 9 | XJ13 | Huocheng, Xinjiang | 5^t^+10^t^ | - |
| 10 | XJ14 | Huocheng, Xinjiang | 5^t^+10^t^ | - |
| 11 | XJ15 | Huocheng, Xinjiang | 2^t^+10^t^ | - |
| 12 | XJ16 | Yining, Xinjiang | 2^t^+10^t^ | - |
| 13 | XJ17 | Yining, Xinjiang | 2^t^+10^t^ | - |
| 14 | XJ18 | Yining, Xinjiang | 5^t^+10^t^ | - |
| 15 | XJ19 | Yining, Xinjiang | 5^t^+10^t^ | - |
| 16 | XJ20 | Yining, Xinjiang | 5^t^+10^t^ | - |
| 17 | XJ21 | Yining, Xinjiang | 2^t^+10^t^ | - |
| 18 | XJ23 | Yining, Xinjiang | 5^t^+10^t^ | - |
| 19 | XJ24 | Yining, Xinjiang | 5^t^+10^t^ | - |
| 20 | XJ25 | Yining, Xinjiang | 2^t^+10^t^ | - |
| 21 | XJ28 | Nilka, Xinjiang | 5^t^+10^t^ | - |
| 22 | XJ29 | Gongliu, Xinjiang | 5^t^+10^t^ | - |
| 23 | XJ30 | Gongliu, Xinjiang | 2^t^+10^t^ | - |
| 24 | XJ31 | Gongliu, Xinjiang | 2^t^+10^t^ | - |
| 25 | XJ32 | Gongliu, Xinjiang | 2^t^+10^t^ | - |
| 26 | XJ35 | Gongliu, Xinjiang | 5^t^+10^t^ | - |
| 27 | XJ38 | Gongliu, Xinjiang | 2^t^+10^t^ | - |
| 28 | XJ39 | Gongliu, Xinjiang | 2^t^+10^t^ | - |
| 29 | XJ40 | Gongliu, Xinjiang | 2^t^+10^t^ | - |
| 30 | XJ41 | Gongliu, Xinjiang | 2^t^+10^t^ | - |
| 31 | XJ42 | Gongliu, Xinjiang | 2^t^+10^t^ | - |
| 32 | XJ43 | Gongliu, Xinjiang | 2^t^+10^t^ | - |
| 33 | XJ44 | Gongliu, Xinjiang | 2^t^+10^t^ | - |
| 34 | XJ45 | Gongliu, Xinjiang | 5^t^+10^t^ | - |
| 35 | XJ46 | Gongliu, Xinjiang | 2^t^+10^t^ | - |
| 36 | XJ47 | Gongliu, Xinjiang | 5^t^+10^t^ | - |
| 37 | XJ48 | Gongliu, Xinjiang | 2^t^+10^t^ | - |
| 38 | XJ49 | Gongliu, Xinjiang | 5^t^+10^t^ | - |
| 39 | XJ50 | Gongliu, Xinjiang | 2^t^+10^t^ | - |
| 40 | XJ51 | Gongliu, Xinjiang | 2^t^+10^t^ | - |
| 41 | XJ52 | Gongliu, Xinjiang | 2^t^+10^t^ | - |
| 42 | XJ53 | Gongliu, Xinjiang | 2^t^+10^t^ | - |
| 43 | XJ54 | Gongliu, Xinjiang | 5^t^+10^t^ | - |
| 44 | XJ55 | Gongliu, Xinjiang | 5^t^+10^t^ | - |
| 45 | XJ56 | Gongliu, Xinjiang | 5^t^+10^t^ | - |
| 46 | XJ58 | Gongliu, Xinjiang | 2^t^+10^t^ | - |
| 47 | XJ59 | Gongliu, Xinjiang | 2^t^+10^t^ | - |
| 48 | XJ60 | Gongliu, Xinjiang | 2^t^+10^t^ | - |
| 49 | XJ61 | Gongliu, Xinjiang | 2^t^+10^t^ | - |
| 50 | XJ62 | Gongliu, Xinjiang | 2^t^+10^t^ | - |
| 51 | XJ64 | Gongliu, Xinjiang | 2^t^+10^t^ | - |
| 52 | XJ65 | Xinyuan, Xinjiang | 2^t^+10^t^ | - |
| 53 | XJ66 | Xinyuan, Xinjiang | 2^t^+10^t^ | - |
| 54 | XJ67 | Xinyuan, Xinjiang | 2^t^+10^t^ | - |
| 55 | XJ69 | Xinyuan, Xinjiang | 2^t^+10^t^ | - |
| 56 | XJ70 | Xinyuan, Xinjiang | 2^t^+10^t^ | - |
| 57 | XJ71 | Xinyuan, Xinjiang | 2^t^+10^t^ | - |
| 58 | XJ72 | Xinyuan, Xinjiang | 2^t^+10^t^ | - |
| 59 | XJ73 | Xinyuan, Xinjiang | 5^t^+10^t^ | - |
| 60 | XJ74 | Xinyuan, Xinjiang | 2^t^+10^t^ | - |
| 61 | XJ75 | Xinyuan, Xinjiang | 2^t^+10^t^ | - |
| 62 | XJ76 | Xinyuan, Xinjiang | 2^t^+10^t^ | - |
| 63 | XJ77 | Xinyuan, Xinjiang | 2^t^+10^t^ | - |
| 64 | XJ78 | Xinyuan, Xinjiang | 2^t^+10^t^ | - |
| 65 | XJ79 | Xinyuan, Xinjiang | 2^t^+10^t^ | - |
| 66 | XJ80 | Xinyuan, Xinjiang | 2^t^+10^t^ | - |
| 67 | XJ81 | Xinyuan, Xinjiang | 2^t^+10^t^ | - |
| 68 | XJ82 | Xinyuan, Xinjiang | 2^t^+10^t^ | - |
| 69 | XJ83 | Xinyuan, Xinjiang | 2^t^+10^t^ | - |
| 70 | XJ84 | Xinyuan, Xinjiang | 2^t^+10^t^ | - |
| 71 | XJ86 | Xinyuan, Xinjiang | 2^t^+10^t^ | - |
| 72 | XJ87 | Xinyuan, Xinjiang | 2^t^+10^t^ | - |
| 73 | XJ88 | Xinyuan, Xinjiang | 2^t^+10^t^ | - |
| 74 | XJ89 | Xinyuan, Xinjiang | 2^t^+10^t^ | - |
| 75 | XJ90 | Xinyuan, Xinjiang | 2^t^+10^t^ | - |
| 76 | XJ91 | Xinyuan, Xinjiang | 2^t^+10^t^ | - |
| 77 | XJ92 | Xinyuan, Xinjiang | 2^t^+10^t^ | - |
| 78 | XJ94 | Xinyuan, Xinjiang | 2^t^+10^t^ | - |
| 79 | XJ95 | Xinyuan, Xinjiang | 2^t^+10^t^ | - |
| 80 | XJ96 | Xinyuan, Xinjiang | 2^t^+10^t^ | - |
| 81 | XJ97 | Xinyuan, Xinjiang | 2^t^+10^t^ | - |
| 82 | XJ98 | Xinyuan, Xinjiang | 2^t^+10^t^ | - |
| 83 | XJ99 | Xinyuan, Xinjiang | 2^t^+10^t^ | - |
| 84 | XJ100 | Xinyuan, Xinjiang | 2^t^+10^t^ | - |
| 85 | XJ101 | Xinyuan, Xinjiang | 2^t^+10^t^ | - |
| 86 | XJ102 | Xinyuan, Xinjiang | 2^t^+10^t^ | - |
| 87 | XJ103 | Xinyuan, Xinjiang | 2^t^+10^t^ | - |
| 88 | XJ104 | Xinyuan, Xinjiang | 2^t^+10^t^ | - |
| 89 | XJ105 | Xinyuan, Xinjiang | 2^t^+10^t^ | - |
| 90 | XJ106 | Xinyuan, Xinjiang | 2^t^+10^t^ | - |
| 91 | XJ107 | Xinyuan, Xinjiang | 2^t^+10^t^ | - |
| 92 | XJ108 | Xinyuan, Xinjiang | 2^t^+10^t^ | - |
| 93 | XJ110 | Xinyuan, Xinjiang | 2^t^+10^t^ | - |
| 94 | XJ109 | Xinyuan, Xinjiang | 2^t^+10^t^ | - |
| 95 | XJ111 | Xinyuan, Xinjiang | 2^t^+10^t^ | - |
| 96 | XJ112 | Xinyuan, Xinjiang | 2^t^+10^t^ | - |
| 97 | AY1 | Azerbaijan | 5^t^+12^t^ | - |
| 98 | AY2 | Qinghai | 2^t^+12^t^ | - |
| 99 | AY3 | Iran | 5^t^+12^t^ | - |
| 100 | AY4 | Azerbaijan | 2^t^+10^t^ | - |
| 101 | AY5 | Turkmenistan | 2.2^t^+10^t^ | - |
| 102 | AY6 | Turkmenistan | 2^t^+12.5^t^ | - |
| 103 | AY9 | Iran | 2.2^t^+10.2^t^ | - |
| 104 | AY8 | Iran | 5^t^+10^t^ | - |
| 105 | AY10 | Afghanistan | 5.5^t^+12.1^t^ | - |
| 106 | AY12 | Pakistan | 2^t^+10^t^ | - |
| 107 | AY11 | Turkmenistan | 2.1^t^+10.1^t^ | - |
| 108 | AY13 | cauca | 2^t^+10^t^ | - |
| 109 | AY14 | Armenia | 2^t^+10^t^ | - |
| 110 | AY16 | Iran | 5.1^t^+10^t^ | - |
| 111 | AY15 | Turkmenistan | 5^t^+10.2^t^ | - |
| 112 | AY17 | Turkey | 2.2^t^+10^t^ | - |
| 113 | AY18 | Tajikistan | 2^t^+10^t^ | - |
| 114 | AY19 | Afghanistan | 2^t^+10^t^ | - |
| 115 | AY20 | India | 2^t^+10^t^ | - |
| 116 | AY21 | Tajikistan | 2^t^+10^t^ | - |
| 117 | AY22 | Pakistan | 2^t^+10.3^t^ | - |
| 118 | AY23 | Iran | 2^t^+10^t^ | - |
| 119 | AY24 | Pakistan | 2^t^+10.1^t^ | - |
| 120 | AY25 | Afghanistan | 2^t^+10^t^ | - |
| 121 | AY26 | Afghanistan | 2^t^+10^t^ | - |
| 122 | AY27 | Afghanistan | 5^t^+10^t^ | - |
| 123 | AY28 | Azerbaijan | 2^t^+10^t^ | - |
| 124 | AY29 | Azerbaijan | 2^t^+10.2^t^ | - |
| 125 | AY30 | Afghanistan | 2^t^+10^t^ | - |
| 126 | AY31 | Afghanistan | 5^t^+10^t^ | - |
| 127 | AY32 | Turkey | 5.5^t^+12.1^t^ | - |
| 128 | AY33 | Russian | 5^t^+10^t^ | - |
| 129 | AY34 | Tajikistan | 2^t^+12^t^ | - |
| 130 | AY35 | Turkmenistan | 2^t^+10^t^ | - |
| 131 | AY36 | Turkmenistan | 2^t^+12^t^ | - |
| 132 | AY37 | Turkmenistan | 2.1^t^+10.1^t^ | - |
| 133 | AY38 | Western Asia | 5^t^+10.2^t^ | - |
| 134 | AY40 | Azerbaijan | 2^t^+12^t^ | - |
| 135 | AY41 | Iran | 5^t^+12^t^ | - |
| 136 | AY42 | Afghanistan | 2^t^+10^t^ | - |
| 137 | AY43 | Turkmenistan | 2^t^+12^t^ | - |
| 138 | AY44 | Turkey | 2^t^+10^t^ | - |
| 139 | AY45 | Afghanistan | 2^t^+10^t^ | - |
| 140 | AY47 | Afghanistan | 2^t^+10^t^ | - |
| 141 | AY48 | Turkmenistan | 2.1^t^+10.1^t^ | - |
| 142 | AY49 | Iran | 5^t^+12^t^ | - |
| 143 | AY50 | Turkey | 2^t^+10^t^ | - |
| 144 | AY51 | Turkey | 2^t^+10^t^ | - |
| 145 | AY52 | Turkmenistan | 2^t^+10^t^ | - |
| 146 | AY53 | Azerbaijan | 2^t^+12.5^t^ | - |
| 147 | AY54 | Afghanistan | 2^t^+10^t^ | - |
| 148 | AY55 | Western Asia | 5^t^+12.5^t^ | - |
| 149 | AY56 | Afghanistan | 2^t^+10^t^ | - |
| 150 | AY57 | Turkmenistan | 2^t^+10^t^ | - |
| 151 | AY58 | Turkey | 2^t^+10^t^ | - |
| 152 | AY59 | Russian | 2^t^+10^t^ | - |
| 153 | AY60 | Iran | 2^t^+12.5^t^ | - |
| 154 | AY61 | Iran | 2^t^+12.5^t^ | - |
| 155 | AY62 | Turkmenistan | 2^t^+10^t^ | - |
| 156 | AY63 | Pakistan | 2^t^+10^t^ | - |
| 157 | AY64 | Azerbaijan | 2^t^+10^t^ | - |
| 158 | AY65 | Iran | 2^t^+10^t^ | - |
| 159 | AY66 | Iran | 5^t^+10^t^ | - |
| 160 | AY67 | Azerbaijan | 2^t^+10^t^ | - |
| 161 | AY68 | Western Asia | 2^t^+10^t^ | - |
| 162 | AY69 | Turkmenistan | 2^t^+10^t^ | - |
| 163 | AY70 | Turkey | 2^t^+10^t^ | - |
| 164 | AY71 | Portugal | 2^t^+10^t^ | - |
| 165 | AY72 | Georgia | 2^t^+10^t^ | - |
| 166 | AY73 | Azerbaijan | 2.2^t^+10.3^t^ | - |
| 167 | AY75 | Russian | 2^t^+10^t^ | - |
| 168 | AY76 | Turkistan | 2^t^+10^t^ | - |
| 169 | AY77 | Iran | 2^t^+12^t^ | - |
| 170 | AY78 | Afghanistan | 2^t^+10^t^ | - |
| 171 | AY79 | Afghanistan | 2.1^t^+10.1^t^ | - |
| 172 | AY80 | Iran | 2^t^+12.5^t^ | - |
| 173 | AY81 | Turkey | 2^t^+10^t^ | - |

Note: a: 76 (+), 95 (-).

Table S2 Nucleotide sequences of HMW-GS genes obtained in this study

| Subunits | Genes | Sequences |
| --- | --- | --- |
| Dx5^t^ | *transcript66* | atggctaagcggttagtcctctttgtggcggtagtcgtcgccctcgtggctctcaccgtcgctgaaggtgaggcctctgagcaactacagtgtgagcgcgagctccaggagctccaggagcgcgagctcaaggcatgccagcaggtcatggaccagcagctccgagacattagccccgagtgccaccccgtcgtcgtcagcccggtcgcgggacaatacgagcagcaaatcgtggtgccgcccaagggcggatctttctaccccggcgagaccacgccaccgcagcaactccaacaacgtatattttggggaatacctgcactactaaaaaggtattacccaagtgtaacttctccgcagcaggtttcatactatccaggccaagcttctccgcaacggccaggacaaggtcagcagccaggacaagggcaacaatcaggacaaggacaacaagggtactacccaacttctccgcaacagccaggacaatggcaacaaccggaacaagggcaaccagggtactacccaacttctccgcagcaatcaggacaattgcaacaaccagcacaagggcagcaaccaggacaagggcaacaaggtcagcagccaggacaagggcaaccagggtactacccaacttcttcgcagctgcagccaggacaattgcaacaaccagcacaagggcaacaagggcagcaaccaggacaagggcaacaaggtcaacagccaggacaagggcaacaaccaggacaaggacaacaaggtcaacagccaggacaagggcaacaaccaggacaagggcaacaaggtcagcagctcggacaaggacaacaagggtactacccaacttctctgcaacagtcgggacaagggcaaccagggtactacccaacttctctgcagcagctaggacaagggcaatcagggtactacccaacttctccgcagcaaccaggacaagggcagcagccaggacaattgcaacaaccagcacaagggcagcaaccaggacaagggcaacaaggtcggcagccaggacaagggcaacaaggccagcagccaggacaagggcagcaaccgggacaagggcaaccagggtactacccaacttctccgcagcagtcaggacaagggcaaccagggtactacccaacttcttcgcagcagccaacacaatcgcagcaaccaggacaagggcaacaaggtcagcaggtaggacaagggcaacaagctcagcagccaggacaagggcagcaaccgggacaagggcagccagggtactacccaacttctccgcagcagtcaggacaagggcaaccagggtactacctaacttctccgcagcagtcaggacaagggcagcagccaggacaattgcaacaatcagcacaagggcaaaaaggacagcaaccaggacaaggtcaacagccagggcaagggcaacaaggtcagcagccaggacaagggcaacaaggtcagcaaccggggcaagggcagccagggtactacccaacttctccgcagcaatcaggacaagggcaacagccaggacaatggcaacaaccaggacaagggcaaccaggatactacccaacttctccgttgcagccaggacaagggcaaccagggtacgacccaacttctccgcaacagccaggacaagggcagcaaccaggacaattgcaacaaccagcacaagggcaacaagggcagcaactagcacaagggcaacaagggcagcaaccagcacaagtgcaacaagggcagcggccagcacaagggcaacaaggtcagcagccaggacaagggcaacaaggtcagcagctaggacaagggcaacaagggcagcagccaggacaagggcaacaagggcagcaaccagcacaagggcaacaaggtcagcagccaggacaagggcaacaaggtcagcagccaggacaagggcaacaaggtcagcagccaggacaagggcagcaaccgggacaagggcagccatggtactacccaacttctccgcaggagtcaggacaagggcaacagccaggacaatggcaacaaccaggacaagggcaaccagggtactacctaacttctccgttgcagctaggacaagggcaacaagggtactacccaacttctctgcaacaaccaggacaagggcagcaaccaggacaatggcaacaatcaggacaagggcaacattggtactacccaacttctccgcagctgtcaggacaagggcaacggccaggacaatggctgcaaccaggacaagggcaacaagggtactacccaacttctccgcaacaaccaggacaagggcaacaactaggacaatggctgcaaccaggacaagggcaacaagggtactacccaacttctctgcaacagacaggacaagggcagcaatcaggacaagggcaacaaggctactacagctcataccatgttagcgtggagcaccaggcggccagcctaaaggtggcaaaggcgcagcagctcgcggcacagctgccggcaatgtgccggctggagggcggcgacgcattgtcggccagccagtgatag |
| Dx2^t^ | *transcript370* | atggctaagcggttagtcctctttgtggcggtagtcgtcgccctcgtggctctcaccgtcgctgaaggtgaggcctctgagcaactacagtgtgagcgcgagctccaggagctccaggagcgcgagctcaaggcatgccagcaggtcatggaccagcagctccgagacattagccccgagtgccaccccgtcgtcgtcagcccggtcgcgggacaatacgagcagcaaatcgtggtgccgcccaagggcggatctctctaccccggcgagaccacgccaccgcagcaactccaacaacgtatattttggggaatacctgcactactaaaaaggtattacccaagtgtaacttctccgcagcaggtttcattctatccaggccaagcttctccgcaacggccaggacaaggtcagcagccaggacaagggcaacaatcaggacaaggacaacaagggtactacccaacttctccgcaacagccaggacaatggcaacaaccggaacaagggcaaccagggtactacccaacttctccgcagcagccaggacaattgcaacaaccagcacaagggcagcaaccaggacaagggcaacaaggtcagcagccaggacaagggcaaccagggtactacccaacttcttcgcagctgcagccaggacaattgcaacaaccagcacaagggcaacaagggcagcaaccaggacaagggcaacaaggtcaacagccaggacaagggcaacaaccaggacaaggacaacaaggtcaacagccaggacaagggcaacaaccaggacaagggcaacaaggtcagcagctcggacaaggacaacaagggtactacccaacttctctgcaacagtcgggacaagggcaaccagggtactacccaacttctctgcagcagctaggacaagggcaatcagggtactacccaacttctccgcagcaaccaggacaagggcagcagctaggacaattgcaacaaccagcacaagggcagcaaccaggacaagggcaacaaggtcagcagccaggacaagggcaacaaggccagcagccaggacaagggcagcaaccgggacaagggcaaccagggtactacccaacttctccgcagcagtcaggacaagggcaaccagggtactacccaacttcttcgcagcagccaacacaatcgcagcaaccaggacaagggcaacaaggtcagcaggtaggacaagggcaacaagctcagcagccaggacaagggcagcaaccgggacaagggcagccagggtactacccaacttctccgcagcagtcaggacaagggcaaccagggtactacctaacttctccgcagcagtcaggacaagggcagcagccaggacaattgcaacaatcagcacaagggcaaaaaggacagcaaccaggacaaggtcaacagccagggcaagggcaacaaggtcagcagccaggacaagggcaacaaggtcagcaaccggggcaagggcagccagggtactacccaacttctccgcagcaatcaggacaagggcaacagccaggacaatggcaacaaccaggacaagggcaaccaggatactacccaacttctccgttgcagccaggacaagggcaaccagggtacgacccaacttctccgcaacagccaggacaagggcagcaaccaggacaattgcaacaaccagcacaagggcaacaagggcagcaactagcacaagggcaacaagggcagcaaccagcacaagggcaacaaggtcagcagccaggacaagggcaacaaggtcagcagctaggacaagggcaacaaggtcagcagccaggacaagggcaacaagggcagcagccagcacaagggcaacaaggtcagcagccaggacaagggcaacaaggtcagcagccaggacaagggcaacaaggtcagcagccaggacaagggcagcaaccgggacaagggcagccatggtactacccaacttctccgcaggagtcaggacaagggcaacagccaggacaatggcaacaaccaggacaagggcaaccagggtactacctaacttctccgttgcagctaggacaagggcagcaagggtactacccaacttctctgcaacaaccaggacaagggcagcaaccaggacaatggcaacaatcgggacaagggcaacatgggtactacccaacttctccgcagctgtcaggacaagggcaacggccaggacaatggctgcaaccaggacaagggcaacaagggtactacccaacttctccgcaacagtcaggacaagggcaacaactaggacaatggctgcaaccaggacaagggcaacaagggtactacccaacttctctgcaacagacaggacaagggcagcaatcaggacaagggcaacaaggctactacagctcataccatgttagcgtggagcaccaggcggccagcctaaaggtggcaaaggcgcagcagctcgcggcacagctgccggcaatgtgccggctggagggcggcgacgcattgtcggccagccagtgatag |
| Dx2^t^ | *transcript506* | atggctaagcggttagtcctctttgtggcggtagtcgtcgccctcgtggctctcaccgtcgctgaaggtgaggcctctgagcaactacagtgtgagcgcgagctccaggagctccaggagcgcgagctcaaggcatgccagcaggtcatggaccagcagctccgagacattagccccgagtgccaccccgtcgtcgtcagcccggtcgcgggacaatacgagcagcaaatcgtggtgccgcccaagggcggatctttctaccccggcgagaccacgccaccgcagcaactccaacaacgtatattttggggaatacctgcactactaaaaaggtattacccaagtgtaacttctccgcagcaggtttcatactatccaggccaagcttctccgcaacggccaggacaaggtcagcagccaggacaagggcaacaatcaggacaaggacaacaagggtactacccaacttctccgcaacagccaggacaatggcaacaaccggaacaagggcaaccagggtactacccaacttctccgcagcagccaggacaattgcaacaaccagcacaagggcagcaaccaggacaaggacaacaaggtcggcagccaggacaagggcaaccagggtactacccaacttcttcgcagctgcagccaggacaattgcaacaaccagcacaagggcaacaagggcagcaaccaggacaagggcaacaaggtcaacagccaggacaagggcaacaatcaggacaaggacaacaaggtcaacagccaggacaagggcaacaaccaggacaagggcaacaaggtcagcagctcggacaaggacaacaagggtactacccaacttctctgcaacagtcgggacaagggcaaccagggtactacccaacttctctgcagcagctaggacaagggcaatcagggtactacccaacttctccgcagcaaccaggacaagggcagcagccaggacaattgcaacaaccagcacaagggcagcaaccaggacaagggcaacaaggtcagcagccaggacaagggcaacaaggccagcagccaggacaagggcagcaaccgggacaagggcaaccagggtactacccaacttctccgcagcagtcaggacaagggcaaccagggtactacccaacttcttcgcagcagccaacacaatcgcagcaaccaggacaagggcaacaaggtcagcaggtaggacaagggcaacaagctcagcagccaagacaagggcagcaaccgggacaagggcagccagggtactacccaacttctccgcagcagtcaggacaagggcaaccagggtactacctaacttctccgcagcagtcaggacaagggcagcagccaggacaattgcaacaatcagcacaagggcaaaaaggacagcaaccaggacaaggtcaacagccagggcaagggcaacaaggtcagcagccaggacaagggcaacaaggtcagcaaccggggcaagggcagccagggtactacccaacttctccgcagcaatcaggacaagggcaacagccaggacaatggcaacaaccaggacaagggcaaccaggatactacccaacttctccgttgcagccaggacaagggcaaccagggtacgacccaacttctccgcaacagccaggacaagggcagcaaccaggacaattgcaacaaccagcacaagggcaacaagggcagcaactagcacaagggcaacaagggcagcaaccagcacaagtgcaacaagagcagcagccagcacaagggcaacaaggtcagcagccaggacaagggcaacaaggtcagcagctaggacaagggcaacaaggtcagcagccaggacaagggcaacaagggcagcaaccagcacaagggcaacaaggtcagcagccaggacaagggcaacaaggtcagcagccaggacaagggcagcaaccgggacaagggcagccatggtactacccaacttctccgcaggagtcaggacaagggcaacagccaggacaatggcaacaaccaggacaagggcaaccagggtactacctaacttctccgttgcagctaggacaagggcaacaagggtactacccaacttctctgcaacaaccaggacaagggcagcaaccaggacaatggcaacaatcgggacaagggcaacatgggtactacccaacttctccgcagctgtcaggacaagggcaacggccaggacaatggctgcaaccaggacaagggcaacaagggtactacccaacttctccgcaacagtcaggacaagggcaacaactaggacaatggctgcaaccaggacaagggcaacaagggtactacccaacttctctgcaacagacaggacaagggcagcaatcaggacaagggcaacaaggctactacagctcataccatgttagcgtggagcaccaggcggccagcctaaaggtggcaaaggcgcagcagctcgcggcacagctgccggcaatgtgccggctggagggcggcgacgcattgtcggccagccagtgatag |
| Dx5.5^t^ | *transcript533* | atggctaagcggttagtcctctttgtggcggtagtcgtcgccctcgtggctctcaccgtcgctgaaggtgaggcctctgagcaactacagtgtgagcacgagctccaggagctccaggagcgcgagctcaaggcatgccagcaggtcatggaccagcagctccgagacattagccccgagtgccaccccgtcgtcgtcagcccggtcgcgggacaatacgagcagcaaatcgtggtgccgcccaagggcggatctttctaccccggcgagaccacgccaccgcagcaactccaacaacgtatattttggggaatacctacactactaaaaaggtattacccaagtgtaacttctccgcagcaggtttcatactatccaggccaagcttctccgcaacggccaggacaaggtcagcagccaggacaagggcaacaatcaggacaaggacaacaagggtactacccaacttctccgcaacagccaggacaatggcaacaaccggaacaagggcaaccagggtactacccaacttctccgcagcagccaggacaattgcaacaaccagcacaagggcagcaaccaggacaagggcaacaaggtcagcagccaggacaagggcaacaaggccagcagccaggacaagggcagcaaccgggacaagggcaaccagggtactacccaacttctccgcagcagtcaggacaagggcaaccagggtactacccaacttcttcgcagcagccaacacaatcgcagcaaccaggacaagggcaacaaggtcagcaggtaggacaagggcaacaagctcagcagccaggacaagggcagcaaccgggacaagggcagccagggtactacccaacttctccgcagcagtcaggacaagggcaaccagggtactacctaacttctccgcagcagtcaggacaagggcagcagccaggacaattgcaacaatcagcacaagggcaaaaaggacagcaaccaggacaaggtcaacagccagggcaagggcaacaaggtcagcagccaggacaagggcaacaaggtcagcaaccggggcaagggcagccagggtactacccaacttctccgcagcaatcaggacaagggcaacagccaggacaatggcaacaaccaggacaagggcaaccaggatactacccaacttctccgttgcagccagaacaagggcaaccagggtacgacccaacttctccgcaacagccaggacaagggcagcaaccaggacaattgcaacaaccagcacaagggcaacaagggcagcaactagcacaagggcaacaagggcagcaaccagcacaagggcaacaaggtcagcagccaggacaagggcaacaaggtcagcagctaggacaagggcaacaaggtcagcagccaggacaagggcaacaagggcagcagccagcacaagggcaacaaggtcagcagccaggacaagggcaacaaggtcagcagccaggacaagggcaacaaggtcagcagccaggacaagggcagcaaccgggacaagggcagccatggtactacccaacttctccgcaggagtcagaacaagggcaacagccaggacaatggcaacaaccaggacaagggcaaccagggtactacctaacttctccgttgcagctaggacaagggcagcaagggtactacccaacttctctgcaacaaccaggacaagggcagcaaccaggacaatggcaacaatcgggacaagggcaacatgggtactacccaacttctccgcagctgtcaggacaagggcaacggccaggacaatggctgcaaccaggacaagggcaacaaggatactacccaacttctccgcaacagtcaggacaagggcaacaactaggacaatggctgcaaccaggacaagggcaacaagggtactacccaacttctctgcaacagacaggacaagggcagcaatcaggacaagggcaacaaggctactacagctcataccatgttagcgtggagcaccaggcggccagcctaaaggtggcaaaggcgcagcagctcgcggcacagctgccggcaatgtgccggctggagggcggcgacgcattgtcggccagccagtgatag |
| Dx2.2^t^ | *transcript720* | atggctaagcggttagtcctctttgtggcggtagtcgtcgccctcgtggctctcaccgtcgctgaaggtgaggcctctgagcaactacagtgtgagcgcgagctccaggagctccaggagcgcgagctcaaggcatgccagcaggtcatggaccagcagctccgagacattagccccgagtgccaccccgtcgtcgtcagcccggtcgcgggacaatacgagcagcaaatcgtggtgccgcccaagggcggatctttctaccccggcgagaccacgccaccgcagcaactccaacaacgtatattttggggaatacctgcactactaaaaaggtattacccaagtgtaacttctccgcagcaggtttcatactatccaggccaagcttctccgcaacggccaggacaaggtcagcagccaggacaagggcaacaatcaggacaaggacaacaagggtactacccaacttctccgcaacagccaggacaatggcaacaaccggaacaagggcaaccagggtactacccaacttctccgcagcagccaggacaattgcaacaaccagcacaagggcagcaaccaggacaaggacaacaaggtcagcagccaggacaagggcaaccagggtactacccaacttcttcgcagctgcagccaggacaattgcaacaaccagcacaagggcaacaagggcagcaaccaggacaagggcaacaaggtcaacagccaggacaagggcaacaaccaggacaaggacaacaaggtcaacagccaggacaagggcaacaaccaggacaagggcaacaaggtcagcagctcggacaaggacaacaagggtactacccaacttctctgcaacagtcgggacaagggcaaccagggtactacccaacttctctgcagcagctaggacaagggcaatcagggtactacccaacttctccgcagcaaccaggacaagggcagcagccaggacaattgcaacaaccagcacaagggcagcaaccaggacaagggcaacaaggtcagcagccaggacaagggcaacaaggccagcagccaggacaagggcagcaaccgggacaagggcaaccagggtactacccaacttctccgcagcagtcaggacaagggcaaccagggtactacccaacttcttcgcagcagccaacacaatcgcagcaaccaggacaagggcaacaaggtcagcaggtaggacaagggcaacaagctcagcagccaggacaagggcagcaaccgggacaagggcagccagggtactacccaacttctccgcagcagtcaggacaagggcaaccagggtactacctaacttctccgcagcagtcaggacaagggcagcagccaggacaattgcaacaatcagcacaagggcaaaaaggacagcaaccaggacaaggtcaacagccagggcaagggcaacaaggtcagcagccaggacaagggcaacaaggtcagcaaccggggcaagggcagccagggtactacccaacttctccgcagcaatcaggacaagggcaacagccaggacaatggcaacaaccaggacaagggcaaccaggatactacccaacttctccgttgcagccaggacaagggcaaccagggtacgacccaacttctccgcaacagccaggacaagggcagcaaccaggacaattgcaacaaccagcacaagggcaacaagggcagcaaccagcacaagggcaacaagggcagcagctagcacaagtgcaacaaggtcagcagctaggacaagggcaacaaggtcagcagccaggacaagggcaacaagggcagcaaccagcacaagggcaacaaggtcagcagccaggacaagggcaacaaggtcagcagccaggacaagggcagcaaccgggacaagggcagccatggtactacccaacttctccgcaggagtcaggacaagggcaacagccaggacaatggcaacaaccaggacaagggcaaccagggtactacctaacttctccgttgcagctaggacaagggcaacaagggtactacccaacttctctgcaacaaccaggacaagggcagcaaccaggacaatggcaacaatcgggacaagggcaacatgagtactacccaacttctccgcagctgtcaggacaagggcaacggccaggacaatggctgcaaccaggacaagggcaacaagggtactacccaacttctccgcaacagtcaggacaagggcaacaactaggacaatggctgcaaccaggacaagggcaacaagggtactacccaacttctctgcaacagacaggacaagggcagcaatcaggacaagggcaacaaggctactacagctcataccatgttagcgtggagcaccaggcggccagcctaaaggtggcaaaggcgcagcagctcgcggcacagctgccggcaatgtgccggctggagggcggcgacgcattgtcggccagccagtgatag |
| Dx5.1^t^ | *transcript780* | atggctaagcggttagtcctctttgtggcggtagtcgtcgccctcgtggctctcaccgtcgctgaaggtgaggcctctgagcaactacagtgtgagcgcgagctccaggagctccaggagcgcgagctcaaggcatgccagcaggtcatggaccagcagctccgagacattagccccgagtgccaccccgtcgtcgtcagcccggtcgcgggacaatacgagcagcaaatcgtggtgccgcccaagggcggatctttctaccccggcgagaccacgccaccgcagcaactccaacaacgtatattttggggaatacctgcactactaaaaaggtattacccaagtgtaacttctccgcagcaggtttcatactatccaggccaagcttctccgcaacggccaggacaaggtcagcagccaggacaagggcaacaatcaggacaaggacaacaaggatactacccaacttctccgcaacagccaggacaatggcaacaaccggaacaagggcaaccagggtactacccaacttctccgcagcaatcaggacaattgcaacaaccagcacaagggcagcaaccaggacaagggcaacaaggtcagcagccaggacaagggcaaccagggtactacccaacttcttcgcagctgcagccaggacaattgcaacaaccagcacaagggcaacaagggcagcaaccaggacaagggcaacaaggtcaacagccaggacaagggcaacaaccaggacaaggacaacaaggtcaacagccaggacaagggcaacaaccaggacaagggcaacaaggtcagcagctcggacaaggacaacaagggtactacccaacttctctgcaacagtcgggacaagggcaaccagggtactacccaacttctctgcagcagctaggacaagggcaatcagggtactacccaacttctccgcagcaaccaggacaagggcagcagccaggacaattgcaacaaccagcacaagggcagcaaccaggacaagggcaacaaggtcggcagccaggacaagggcaacaaggccagcagccaggacaagggcagcaaccgggacaagggcaaccagggtactacccaacttctccgcagcagtcaggacaagggcaaccagggtactacccaacttcttcgcagcagccaacacaatcgcagcaaccaggacaagggcaacaaggtcagcaggtaggacaagggcaacaagctcagcagccaggacaagggcagcaaccgggacaagggcagccagggtactacccaacttctccgcagcagtcaggacaagggcaaccagggtactacctaacttctccgcagcagtcaggacaagggcagcagccaggacaattgcaacaatcagcacaagggcaaaaaggacagcaaccaggacaaggtcaacagccagggcaagggcaacaaggtcagcagccaggacaagggcaacaaggtcagcaaccggggcaagggcagccagggtactacccaacttctccgcagcaatcaggacaagggcaacagccaggacaatggcaacaaccaggacaagggcaaccaggatactacccaacttctccgttgcagccaggacaagggcaaccagggtacgacccaacttctccgcaacagccaggacaagggcagcaaccaggacaattgcaacaaccagcacaagggcaacaagggcagcaactagcacaagggcaacaagggcagcaaccagcacaagtgcaacaagggcagcggccagcacaagggcaacaaggtcagcagccaggacaagggcaacaaggtcagcagctaggacaagggcaacaagggcagcagccaggacaagggcaacaagggcagcaaccagcacaagggcaacaaggtcagcagccaggacaagggcaacaaggtcagcagccaggacaagggcaacaaggtcagcagccaggacaagggcagcaaccgggacaagggcagccatggtactacccaacttctccgcaggagtcaggacaagggcaacagccaggacaatggcaacaaccaggacaagggcaaccagggtactacctaacttctccgttgcagctaggacaagggcaacaagggtactacccaacttctctgcaacaaccaggacaagggcagcaaccaggacaatggcaacaatcgggacaagggcaacattggtactacccaacttctccgcagctgtcaggacaagggcaacggccaggacaatggctgcaaccaggacaagggcaacaagggtactacccaacttctccgcaacaaccaggacaagggcaacaactaggacaatggctgcaaccaggacaagggcaacaagggtactacccaacttctctgcaacagacaggacaagggcagcaatcaggacaagggcaacaaggctactacagctcataccatgttagcgtggagcaccaggcggccagcctaaaggtggcaaaggcgcagcagctcgcggcacagctgccggcaatgtgccggctggagggcggcgacgcattgtcggccagccagtgatag |
| Dx2.1^t^ | *transcript928* | atggctaagcggttagtcctctttgtggcggtagtcgttgccctcgtggctctcaccgtcgctgaaggtgaggcctctgagcaactacagtgtgagcgcgagctccaggagctccaggagcgcgagctcaaggcatgccagcaggtcatggaccagcagctccgagacattagccccgagtgccaccccgtcgtcgtcagcccggtcgcgggacaatacgagcagcaaatcgtggtgccgcccaagggcggatctttctaccccggcgagaccacgccaccgcagcaactccaacaacgtatattttggggaatacctgcactactaaaaaggtattacccaagtgtaacttctccgcagcaggtttcatactatccaggccaagcttctccgcaacggccaggacaaggtcagcagccaggacaagggcaacaatcaggacaaggacaacaagggtactatccaacttctccgcaacagccaggacaatggcaacaaccggaacaagggcaaccagggtactacccaacttctccacagcagccaggacaattgcaacaaccagcacaagggcagcaaccaggacaaggacaacaaggtcagcagccaggacaagggcaaccagggtactacccaacttcttcgcagctgcagccaggacaattgcaacaaccagcacaagggcaacaagggcagcaaccaggacaagggcaacaaggtcaacagccaggacaagggcaacaaccaggacaaggacaacaaggtcaacagccaggacaagggcaacaaccaggacaagggcaacaaggtcagcagctcggacaaggacaacaagggtactacccaacttctctgcaacagtcgggacaagggcaaccagggtactacccaacttctctgcagcagctaggacaagggcaatcagggtactacccaacttctccgcagcaaccaggacaagggcagcagccaggacaattgcaacaaccagcacaagggcagcaaccaggacaagggcaacaaggtcagcagccaggacaagggcaacaaggccagcagccaggacaagggcagcaaccgggacaagggcaaccagggtactacccaacttctccgcagcagtcaggacaagggcaaccagggtactacccaacttcttcgcagcagccaacacaatcgcagcaaccaggacaagggcaacaaggtcagcaggtaggacaagggcaacaagctcagcagccaggacaagggcagcaaccgggacaagggcagccagggtactacccaacttctccgcagcagtcaggacaagggcaaccagggtactacctaacttctccgcagcagtcaggacaagggcagcagccaggacaattgcaacaatcagcacaagggcaaaaaggacagcaaccaggacaaggtcaacagccagggcaagggcaacaaggtcagcagccaggacaagggcaacaaggtcagcaaccggggcaagggcagccagggtactacccaacttctccgcagcaatcaggacaagggcaacagccaggacaatggcaacaaccaggacaagggcaaccaggatactacccaacttctccgttgcagccaggacaagggcaaccagggtacgacccaacttctccgcaacagccaggacaagggcagcaaccaggacaattgcaacaaccagcacaagggcaacaagggcagcaactagcacaagggcaacaagggcagcaaccagcacaagtgcaacaagagcagcagccagcacaagggcaacaaggtcagcagctaggacaagggcaacaaggtcagcagccaggacaagggcaacaagggcagcaaccagcacaagggcaacaaggtcagcagccaggacaagggcaacaaggtcagcagccaggacaagggcagcaaccgggacaagggcagccatggtactacccaacttctccgcaggagtcaggacaagggcaacagccaggacaatggcaacaaccaggacaagggcaaccagggtactacctaacttctccgttgcagctaggacaagggcaacaagggtactacccaacttctctgcaacaaccaggacaagggcagcaaccaggacaatggcaacaatcgggacaagggcaacatgagtactacccaacttctccgcagctgtcaggacaagggcaacggccaggacaatggctgcaaccaggacaagggcaacaagggtactacccaacttctccgcaacagtcaggacaagggcaacaactaggacaatggctgcaaccaggacaagggcaacaagggtactacccaacttctctgcaacagacaggacaagggcagcaatcaggacaagggcaacaaggctactacagctcataccatgttagcgtggagcaccaggcggccagcctaaaggtggcaaaggcgcagcagctcgcggcacagctgccggcaatgtgccggctgga-gggcggcgacgcattgtcggccagccagtga |
| Dy12^t^ | *transcript51* | atggctaagcggctggtcctctttgcggcagtagtcatcgccctcgtggctctcaccaccgctgaaggtgaggcctctaggcaactacagtgtgagcgcgagctccaggagagctcgcttgaggcatgccggcaggtcgtggaccaacagttggccggtcggctgccatggagcacggggctccagatgcgatgctgccagcagctccgagatgttagcgccaagtgccgctctgtcgccgtcagccaagtcgcaagacaatatgagcaaactgtggtgccgcccaagggcggatccttctaccctggtgagaccacgccactgcagcaactccaacaaggaatattttggggaacatcttcacaaacagtacaagggtattacccaagcgtaacttctcctcggcaggggtcatattatccaggccaagcttctccacaacagccaggacaagggcaacagcctggcaaatggcaagaaccaggacaagggcaacaatggtactacccaacttctctgcagcagccaggacaagggcaacagataggaaaagggaaacaagggtactacccaacttctctgcagcaaccaggacaagggcaacaaataggacaagggcaacaagggtactacccaacttctccgcagcacacaggacaaaggcaacaaccagtacaagggcaacaaataggacaagggcaacaaccagaacaagggcaacaaccaggacaatggcaacaagggtactatccaacttctccacagcagctaggacaagggcaacaaccaggacaatggcaacaatcaggacaagggcaacaagggcactacccaacttctctacaacagccaggacaagggcaacaagggcattacctagcttctcagcagcagccagcacaagggcaacaagggcactacccaacttctcagcagcagccaggacaagggcaacaagggcactacccagcttctcagcagcagccaggacaagggcaacaagggcactacccagcttctcagcaagagccaggacaagggcaacaagggcaaatcccagcttctcaacagcagccaggacaagggcaacaagggcactacccagcttctctgcagcaaccaggacaacaagggcattacccaacttctctacagcagctaggacaagggcaacaaataggacagccaggacaaaagcaacaaccaggacaagggcaacagccagaacaagagcaacaaccaggacaagggcaacaaggatactatccaacttctctgcagcagccaggacaagggcaacagcaaggacaagggcaacaagggtactacccaacttctctccagcagccaggacaagggcaacaagggcactacccagcttctctgcagcagccaggacaaggacagccaggacaaaggcaacaaccaggacaagggcaacatccagaacaagggcaacaaccaggacaagggcaacaagggtactatccaacttctccacagcagccaggacaagggcaacaactaggacaagggcaacaagggtactacccaacttctccgcagcagccaggacaagggcaacaaccaggacaagggcaacaagggcactgcccaacgtccccgcagcagacaggacaagcgcaacaaccaggacaaggccaacaaataggacaagtgcaacaaccaggacaagggcaacaagggtactacccaacttctctgcagcagcctggacaagggcaacagtcaggacaagggcaacagtcaggacaaggacaccaaccaggacaagggcagcaatcaggacaagagcaacaaggctacgacagcccataccatgttagcgcagagcagcaagcggccagcccaatggtggcaaaggcgcagcagcccgcgacacagctgccgacagtgtgtcggatggaggggggcgacgcattgtcggctagccagtgatag |
| Dy10.1^t^ | *transcript67* | atggttaagcggctggtcctctttgcggcagtagtcatcgccctcgtggctctcaccaccgctgaaggtgaggcctctaggcaactacagtgtgagcgcgagctccaggagagctcgcttgaggcatgccggcaggtcgtggaccaacagttggccggtcggctgccatggagcacggggctccagatgcgatgctgccagcagctccgagatgttagcgccaagtgccgctccgtcgccgtcagccaagtcgcaagacaatatgagcaaactgtggtgccgcccaagggcagatccttctaccctggtgagaccacgccactgcagcaactccaacaaggaatattttggggaacatcttcacaaacagtacaagggtattacccaagcgtaacttctcctcggcaggggtcatattatccaggccaagcttctccacaacagccaggacaagggcaacaacctggcaaatggcaagaaccaggacaagggcaacaatggtactacccaacttctctgcagcagccaggacaagggcaacagataggaaaagggcaacaagggtactacccaacttctctgcagcagccaggacaagggcaacaaataggacaagggcaacaagggtactacccaacttctccgcagcacacaggacaaaggcaacaaccagtacaagggcaacaaataggacaagggcaacaaccagaacaagagcaacaaccaggacaatggcaacaagggtactatccaacttctccacagcagctaggacaagggcaacaaccaggacaatggcaacaatcaggacaagggcaacaagggcactacccaacttctctacaacagccaggacaagggcaacaagggcattacctagcttctcagcagcagccaggacaagggcaacaagggcactacccagcttctcagcagcagccaggacaagggcaacaagggcactacccagcttctcagcagcagccaggacaagggcaacaagggcactacccagcttctcagcaagagccaggacaagggcaacaagggcaaatcccagcttctcagcagcagccaggacaagggcaacaagggcactacccagcttctctgcagcaaccaggacaagggcaacaagggcattacccaacttctctacagcagctaggacaagggcaacaaataggacagccaggacaagggcaacaaacagaacaagggcaacaaatagaacaaccaggacaagggcaacaaacaggacaagggcaacagccagaacaagagcaacaaccaggacaagggcaacaaggatactatccaacttctctgcagcagccaggacaagggcaacagcaaggacaagggcaacaagggtactacccaacttctctccagcagccaggacaagggcaacaagggcactatccagcttctctgcagcagccaggacaaggacaaccaggacaaaggcaacaaccaggacaagggcaacatccagaacaagggcaacaaccaggacaagggcaacaagggtactatccaacttctccacagcagccaggacaagggcaacaactaggacaagggcaacaagggtactacccaacttctccgcagcagccaggacaagggcaacaagggcactgcccaacgtccccgcagcagacaggacaagcgcagcaaccaggacaaggccaacaaataggacaagtgcaacaaccaggacaagggcaacaagggtactacccaacttctctgcagcagcctggacaagggcaacagtcaggacaagggcaacagtcaggacaaggacaccaaccaggacaagggcagcaattaggacaagagcaacaaggctacgacagcccataccatgttagcgcagagcagcaagcggccagcccaatggtggcaaaggcgcagcagcccgcgacacagctgccgacagcgtgtcggatggaggggggcgacgcattgtcggctagccagtgatag |
| Dy12.5^t^ | *transcript361* | atggctaagcggctggtcctctttgcggcagtagtcatcgccctcgtggctctcaccaccgctgaaggtgaggcctctaggcaactacagtgtgagcgcgagctccaggagagctcgcttgaggcatgccggcaggtcgtggaccaacagttggccggtcggctgccatggagcacggggctccagatgcgatgctgccagcagctccgagatgttagcgccaagtgccgctccgtcgccgtcagccaagtcgcaagacaatatgagcaaactgtggtgccgcccaagggcggatccttctaccctggtgagaccacaccactgcagcaactccaacaaggaatattttggggaacatcttcacaaacagtacaagggtattacccaagcgtaacttctcctcggcaggggtcatattatccaggccaagcttctccacaacagccaggacaagggcaacagcctggcaaatggcaagaaccaggacaagggcaacaatggtactacccaacttctctgcagcagccaggacaagggcaacagataggaaaagggcaacaagggtactacccaacttctctgcagcagccaggacaagggcaacaaataggacaagggaaacaagggtactacccaacttctccgcagcacacaggacaaaggcaacaaccagtacaagggcaacaaccagaacaagggcaacaaccaggacaatggcaacaagggtactatccaacttctccacagcagctaggacaagggcaacaaccaggacaatggcaacaatcaggacaagggcaacaagggcactacccaacttctcagcaacagccaggacaagggcaacaagggcactacccagcttctcagcagcagccaggacaagggcaacaagggcactacccagcttctcagcagcagccaggacaagggcaacaagggcactacccagcttctcagcagcagccaggacaagggcaacaagggcactacccagcttctctacagcaaccaggacaagggcaacaagggcattacccaacttctctacagcagctaggacaagggcaacaaacaggacaaccaggacaaaagcaacaactaggacaagggcaacaaacaggacatgggcaacagccagaacaagagcaacaacaaggacaagggcaacaaggatactatccaacttctctgcagcagccaggacaagggcaacagcaaggacaagggcaacaagggtactacccaacttctctccagcagccaggacaagggcaacaagggcactacccagcttctctgcagcagccaggacaaggacagccaggacaaaggcaacaaccaggacaagggcaacatccagaacaagggcaacaaccaggacaagggcaacaagggtactatccaacttctccacagcagccaggacaagggcaacaactaggacaagggcaacaagggtactacccaacttctccgcagcagccaggacaagggcaacaaccaggacaagggcaacaagggcactgcccaacgtctctgcagcagacaggacaagcacaacaatcaggacaaggccaacaaataggacaagtgcaacaaccaggacaagggcaacaagggtactacccaacttctctgcagcagcttggacaagggcaacagtcaggacaagggcaacaatcaggacaaggacaccaaccaggacaagggcaacaatcaggacaagagcaacaaggctacgacggcccataccatgttagcgcggagcagcaagcggctagcccaatggtggcaaaggcgcagcagcccgcgacacagctgccgacagtgtgtcggatggaggggggcgacgcattgtcagctagccagtgatag |
| Dy10.2^t^ | *transcript447* | atggttaagcggctggtcctctttgcggcagtagtcatcgccctcgtggctctcaccaccgctgaaggtgaggcctctaggcaactacagtgtgagcgcgagctccaggagagctcgcttgaggcatgccggcaggtcgtggaccaacagttggccggtcggctgccatggagcacggggctccagatgcgatgctgccagcagctccgagatgttagcgccaagtgccgctccgtcgccgtcagccaagtcgcaagacaatatgagcaaactgtggtgccgcccaagggcggatccttctaccctggtgagaccacgccactgcagcaactccaacaaggaatattttggggaacatcttcacaaacagtacaagggtattacccaagcgtaacttctcctcggcaggggtcatattatccaggccaagcttctccacaacagccaggacaagggcaacagcctggcaaatggcaagaaccaggacaagggcaacaatggtactacccaacttctctgcagcagccaggacaagggcaacagataggaaaagggcaacaagggtactacccaacttctctgcagcagccaggacaagggcaacaaataggacaagggcaacaagggtactacccaacttctccgcagcacacaggacaaaggcaacaaccagtacaagggcaacaaataggacaagggcaacaaccagaacaagagcaacaaccaggacaatggcaacaagggtactatccaacttctccacagcagctaggacaagggcaacaaccaggacaatggcaacaatcaggacaagggcaacaagggcactaccaaacttctctacaacagccaggacaagggcaacaagggcattacctagcttctcagcagcagccaggacaagggcaacaagggcactacccagcttctcagcagcagccaggacaagggcaacaagggcactacccagcttctcagcagcaaccaggacaagggcaacaagggcactacccagcttctcagcaagagccaggacaagggcaacaagggcaaatcccagcttctcagcagcagccaggacaagggcaacaagggcactacccagcttctctgcagcaaccaggacaagggcaacaagggcattacccaacttctctacaacagctaggacaagggcaacaaataggacagccaggacaaaagcaacaaccaggacaagggcaacaaacaggacaagggcaacagccagaacaagagcaacaaccaggacaagggcaacaaggatactatccaacttctctgcagcagccaggacaagggcaacagcaaggacaagggcaacagcaaggacaagggcaacaagggtactacccaacttctctccagcagccaggacaagggcaacaagggcactacccagcttctctgcagcagccaggacaaggacaaccaggacaaaggcaacaaccaggacaagggcaacatccagaacaagggcaacaaccaggacaagggcaacaagggtactatccaacttctccacagcagccaggacaagggcaacaactaggacaagggcaacaagggtactacccaacttctccgcagcagcgaggacaagggcaacaagggcactgcccaacgtccccgcagcagacaggacaagcgcaacaaccaggacaaggccaacaaataggacaagtgcaacaaccaggacaagggcaacaagggtactacccaacttctctgcagcagcctggacaagggcaacagtcaggacaagggcaacagtcaggacaaggacaccaaccaggacaagggcagcaattaggacaagagaaacaaggctacgacagcccataccatgttagcgcagagcagcaagcggccagcccaatggtggcaaaggcgcagcagcccgcgacacagctgccgacagtgtgtcggatggaggggggcgacgcattgtcggctagccagtgatag |
| Dy10^t^ | *transcript637* | atggctaagcggctggtcctctttgcggcagtagtcatcgccctcgtggctctcaccaccgctgaaggtgaggcctctaggcaactacagtgtgagcgcgagctccaggagagctcgcttgaggcatgccggctggtcgtggaccaacagttggccggtcggctgccatggagcacggggctccagatgcgatgctgccagcagctccgagatgttagcgccaagtgccgctctgtcgccgtcagccaagtcgcaagacaatatgagcaaactgtggtgccgcccaagggcggatccttctaccctggtgagaccacgccactgcagcaactccaacaaggaatattttggggaacatcttcacaaacagtacaagggtattacccaagcgtaacttctcctcggcaggggtcatattatccaggccaagcttctccacaacagccaggacaagggcaacagcctggcaaatggcaagaaccaggacaagggcaacaatggtactacccaacttctctgcagcagccaggacaagggcaacagataggaaaagggaaacaagggtactacccaacttctctgcagcaaccaggacaagggcaacaaataggacaagggcaacaagggtactacccaacttctccgcagcacacaggacaaaggcaacaaccagtacaagggcaacaaataggacaagggcaacaaccagaacaagggcaacaaccaggacaatggcaacaagggtactatccaacttctccacagcagctaggacaagggcaacaaccaggacaatggcaacaatcaggacaagggcaacaagggcactacccaacttctctacaacagccaggacaagggcaacaagggcattacctagcttctcagcagcagccagcacaagggcaacaagggcactacccaacttctcagcagcagccaggacaagggcaacaagggcactacccagcttctcagcagcagccaggacaagggcaacaagggcactacccagcttctcagcaagagccaggacaagggcaacaagggcaaatcccagcttctcaacagcagccaggacaagggcaacaagggcactacccagcttctctacagcaaccaggacaacaagggcattacccaacttctctacagcagctaggacaagggcaacaaataggacagccaggacaaaagcaacaaccaggacaagggcaacaaacaggacaagggcaacagccagaacaagagcaacaaccaggacaagggcaacaaggatactatccaacttctctgcagcagccaggacaagggcaacagcaaggacaagggcaacaagggtactacccaacttctctccagcagccaggacaagggcaacaagggcactacccagcttctctgcagcagccaggacaaggacagccaggacaaaggcaacaaccaggacaagggcaacatccagaacaagggcaacaaccaggacaagggcaacaagggtactatccaacttctccacagcagccaggacaagggcaacaactaggacaagggcaacaagggtactacccaacttctccgcagcagccaggacaagggcaacaaccaggacaagggcaacaagggcactgcccaacgtccccgcagcagacaggacaagcgcaacaaccaggacaaggccaacaaataggacaagtgcaacaaccaggacaagggcaacaagggtactacccaacttctctgcagcagcctggacaagggcaacagtcaggacaagggcaacagtcaggacaaggacaccaaccaggacaagggcagcaatcaggacaagagcaacaaggctacgacagcccataccatgttagcgcagagcagcaagcggccagcccaatggtggcaaaggcgcagcagcccgcgacacagctgccgacagtgtgtcggatggaggggggcgacgcattgtcggctagccagtgatag |
| Dy12.1^t^ | *transcript690* | atggctaagcggctggtcctctttgcggcagtagtcatcgccctcgtggctctcaccactgctgaaggtgaggcctctaggcaactacagtgtgagcgcgagctccaggagagctcgcttgaggcatgccggcaggttgtggaccaacagttggccggtcggctgccatggagcacggggctccagatgcgatgctgccagcagctccgagatgttagcgccaagtgccgctccgtcgccatcagccaagtcgcaagacaatatgagcaaactgtggtgccgcccaagggcggatccttctaccctggtgagaccacgccactgcagcaactccaacaaggaatattttggggaacatcttcacaaacagtacaagggtattacccaggcgtaacttctcctcggcaggggtcatattatccaggccaagcttctccacaacagccaggacaagggcaacagcctggcaaatggcaagaaccaggacaagggcaacaatggtactacccaacttctttgcagcagccaggacaagggcaacagataggaaaagggcaacaagggtactacccaacttctctgcagcagccaggacaagggcaacaagggtactacccaacttctctgcagcacacaggacaaaggcaacaaccagtacaagggcaacaaccagaacaagggcaacaaccaggacaatggcaacaagggtactatccaacttctccacaacagctaggacaagggcaacaaccaggacaatggcaacaatcaggacaagggcaacaagggcactacccaacttctctacaacagccaggacaagggcaacaagggcattacctagcttctcagcagcagccaggacaagggcaacaagggcactacccagcttctcagcagcagccaggacaagggcaacaagggcactacccagcttctcagcagcagccaggacaagggcaacaagggcactacccagcttctcagcaagagccaggacaagggcaacaagggcaaatcccagcttctcagcagcagccaggacaagggcaacaagggcactacccagcttctctgcagcaaccaggacaagggcaacaagggcattacccaacttctctacagcagctaggacaagggcaacaaacaggacagccaggacaaaagcaacaaccaggacaagggcaacaaacaggacaagggcaacagccagaacaagagcaacaaccaggacaagggcaacaaggatactatccaacttctctgcagcagccaggacaagggcaacagcaaggacaagggcaacaagggtactacccaacttctctccagcagccaggacaagggcaacaagggcactacccagcttctctgcagcagccaggacaaggacagccaggacaaaggcaacaaccaggacaagggcaacatccagaacaagggaaacaaccaggacaagggcaacaagggtactatccaacttctccacagcagccaggacaagggcaacaactaggacaagggcaacaagggtactacccaacttctccgcagcagccaggacaagggcaacaaccaggacaagggcaacaagggcactgcccaacgtccccgcagcagtcaggacaagcgcaacaaccaggacaaggccaacaaataggacaagtgcaacaaccaggacaagggcaacaagggtactacccaacttctctgcagcagcctggacaagggcaacaatcaggacaagggcaacagtcaggacaaggacaccaaccaggacaagggcagcaatcaggacaagagcaacaaggctacgacagcccataccatgttagcgcagagcagcaagcggccagcccaatggtggcaaaggcgcagcagcccgcgacacagctgccgacagtgtgtcggatggaggggggcgacgcattgtcggctagccagtgatag |
| Dy10.3^t^ | *transcript754* | atggttaagcggctggtcctctttgcggcagtagtcatcgccctcgtggctctcaccaccgctgaaggtgaggcctctaggcaactacagtgtgagcgcgagctccaggagagctcgcttgaggcatgccggcaggtcgtggaccaacagttggccggtcggctgccatggagcacggggctccagatgcgatgctgccagcagctccgagatgttagcgccaagtgccgctccgtcgccgtcagccaagtcgcaagacaatatgagcaaactgtggtgccgcccaagggcggatccttctaccctggtgagaccacgccactgcagcaactccaacaaggaatattttggggaacatcttcacaaacagtacaagggtattacccaagcgtaacttctcctcggcaggggtcatattatccaggccaagcttctccacaacagccaggacaagggcaacaacctggcaaatggcaagaaccaggacaagggcaacaatggtactacccaacttctctgcagcagccaggacaagggcaacagataggaaaagggcaacaagggtactacccaacttctctgcagcagccaggacaagggcaacaaataggacaagggcaacaagggtactacccaacttctccgcagcacacaggacaaaggcaacaaccagtacaagggcaacaaataggacaagggcaacaaccagaacaagagcaacaaccaggacaatggcaacaagggtactatccaacttctccacagcagctaggacaagggcaacaaccaggacaatggcaacaatcaggacaagggcaacaagggcactacccaacttctctacaacagccaggacaagggcaacaagggcattacctagcttctcagcagcagccaggacaagggcaacaagggcactacccagcttctcagcagcagccaggacaagggcaacaagggcactacccagcttctcagcagcagccaggacaagggcaacaagggcactacccagcttctcagcaagagccaggacaagggcaacaagggcaaatcccagcttctcagcagcagccaggacaagggcaacaagggcactacccagcttctctgcagcaaccaggacaagggcaacaagggcattacccaacttctctacagcagctaggacaagggcaacaaataggacagccaggacaaaagcaacaaccaggacaagggcaacaaacaggacaagggcaacagccagaacaagagcaacaaccaggacaagggcaacaaggatactatccaacttctctgcagcagccaggacaagggcaacagcaaggacaagggcaacaagggtactacccaacttctctccagcagccaggacaagggcaacaagggcactacccagcttctctgcagcagccaggacaaggacaaccaggacaaaggcaacaaccaggacaagggcaacatccagaacaagggcaacaaccaggacaagggcaacaagggtactatccaacttctccacagcagccaggacaagggcaacaactaggacaagggcaacaagggtactacccaacttctccgcagcagccaggacaagggcaacaagggcactgcccaacgtccccgcagcagacaggacaagcgcaacaaccaggacaaggccaacaaataggacaagtgcaacaaccaggacaagggcaacaagggtactacccaacttctctgcagcagcctggacaagggcaacagtcaggacaagggcaacagtcaggacaaggacaccaaccaggacaagggcagcaattaggacaagagcaacaaggctacgacagcccataccatgttagcgcagagcagcaagcggccagcccaatggtggcaaaggcgcagcagcccgcgacacagctgccgatagcgtgtcggatggaggggggcgacgcattgtcggctagccagtgatag |


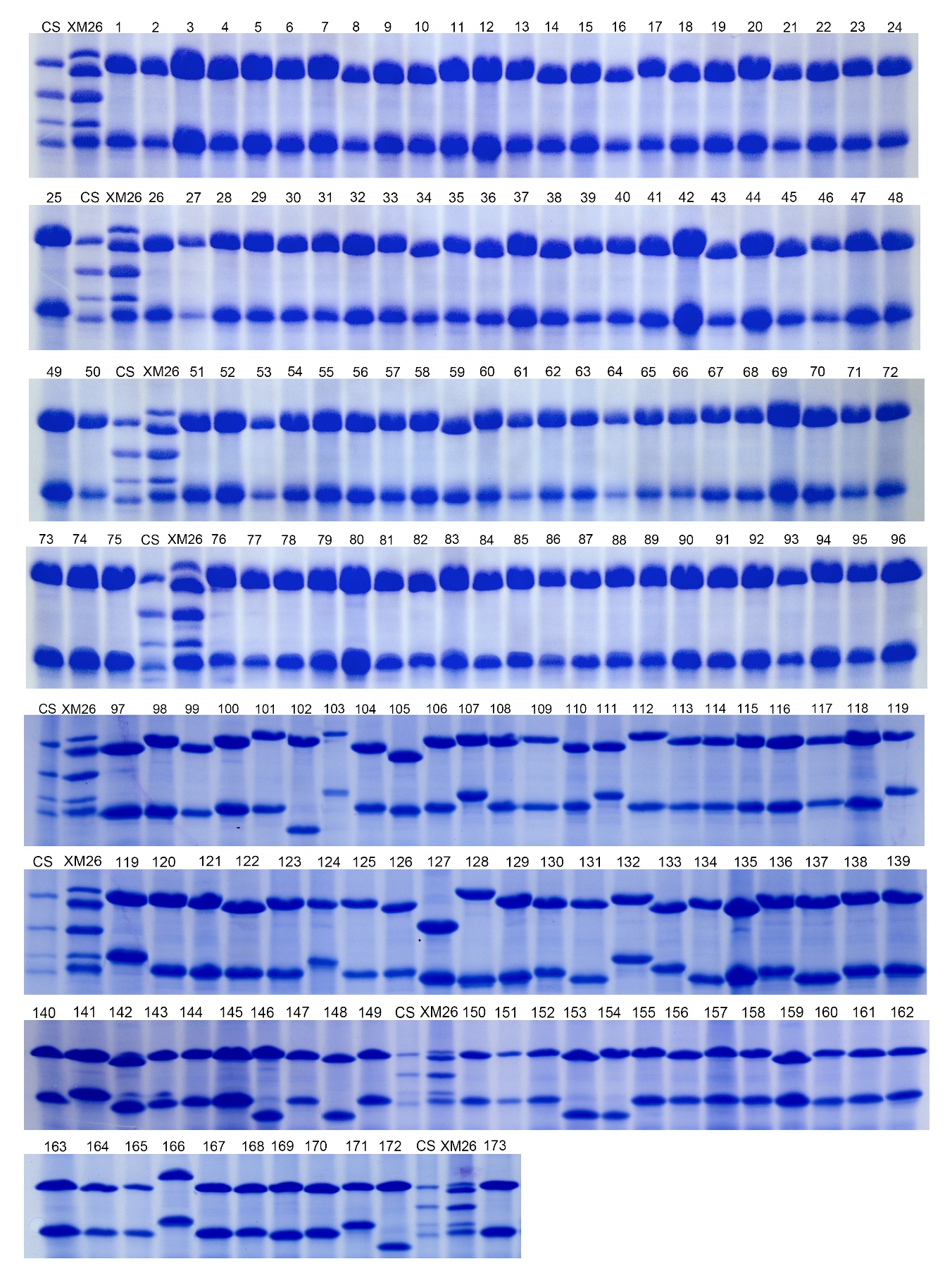


Figure S1 SDS-PAGE patterns of HMW-GS in 173 *Ae.tauschii* accessions from the Middle East, Central Asia and Xinjiang. (CS: Chinese spring; XM26: Xinmai 26; 1-173: Consistent with the serial numbers in Table S1.)


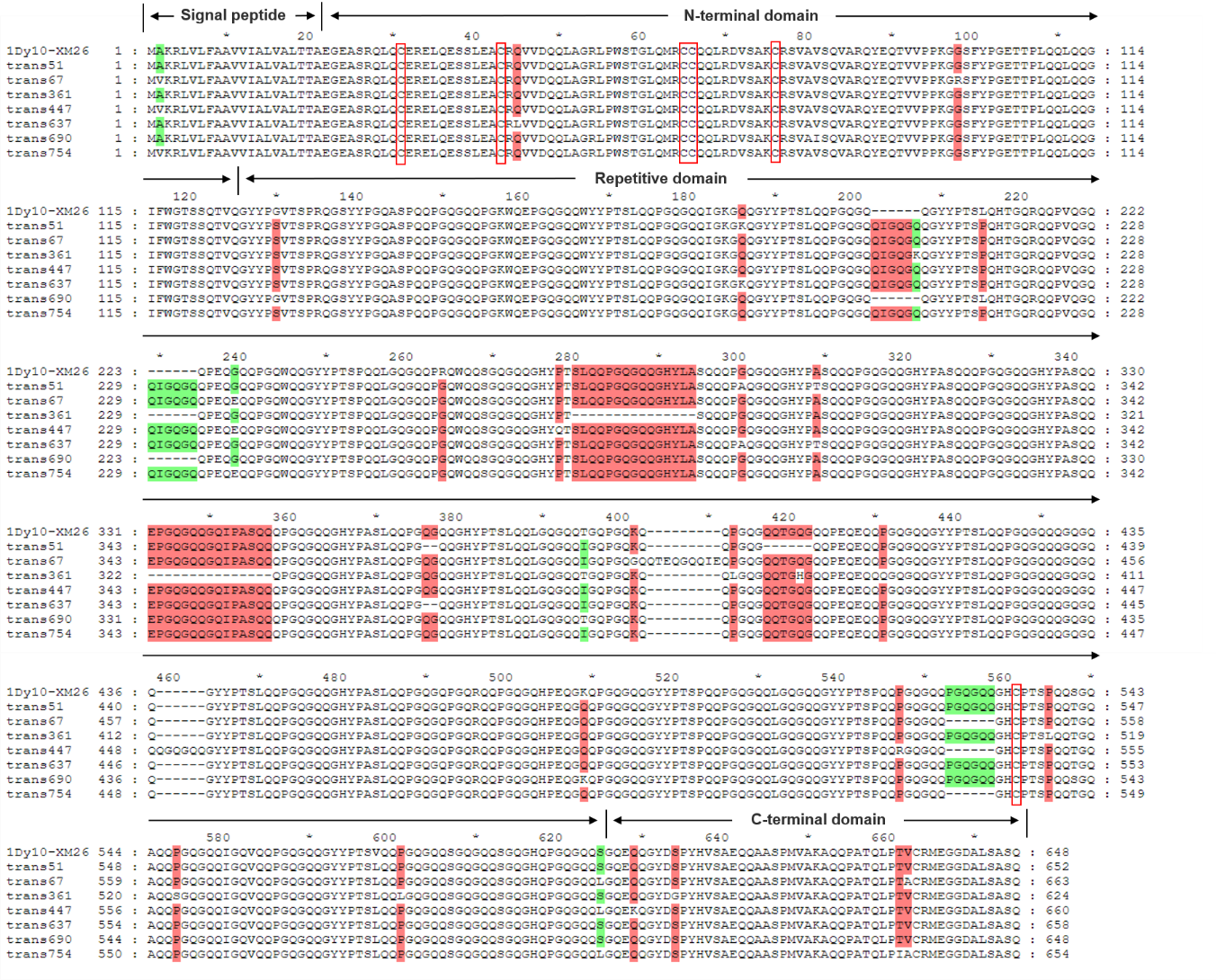


Figure S2 Sequence alignment of the 1Dy10 subunit in Xinmai 26 and 7 identified Dy subunit genes from *Ae. tauschii*. The red boxes indicate the conserved cysteine residues.
